# The type III effector RipB from *Ralstonia solanacearum* RS1000 acts as a major avirulence factor in *Nicotiana benthamiana* and other *Nicotiana* species

**DOI:** 10.1111/mpp.12824

**Published:** 2019-06-20

**Authors:** Masahito Nakano, Takafumi Mukaihara

**Affiliations:** ^1^ Research Institute for Biological Sciences, Okayama (RIBS) Yoshikawa, Kibichuo‐cho Okayama 716‐1241 Japan

**Keywords:** disease resistance, effector‐triggered immunity, *Nicotiana* plants, *Ralstonia solanacearum*, type III effector

## Abstract

*Ralstonia solanacearum* is the causal agent of bacterial wilt in solanaceous crops. This pathogen injects approximately 70 effector proteins into plant cells via the Hrp type III secretion system in an early stage of infection. To identify an as‐yet‐unidentified avirulence factor possessed by the Japanese tobacco‐avirulent strain RS1000, we transiently expressed RS1000 effectors in *Nicotiana benthamiana* leaves and monitored their ability to induce effector‐triggered immunity (ETI). The expression of RipB strongly induced the production of reactive oxygen species and the expressions of defence‐related genes in *N. benthamiana*. The *ripB* mutant of RS1002, a nalixidic acid‐resistant derivative of RS1000, caused wilting symptoms in *N. benthamiana*. A pathogenicity test using *R. solanacearum* mutants revealed that the two already known avirulence factors RipP1 and RipAA contribute in part to the avirulence of RS1002 in *N. benthamiana*. The Japanese tobacco‐virulent strain BK1002 contains mutations in *ripB* and expresses a C‐terminal‐truncated RipB that lost the ability to induce ETI in *N. benthamiana*, indicating a fine‐tuning of the pathogen effector repertoire to evade plant recognition. RipB shares homology with *Xanthomonas* XopQ, which is recognized by the resistance protein Roq1. The RipB‐induced resistance against *R. solanacearum* was abolished in *Roq1*‐silenced plants. These findings indicate that RipB acts as a major avirulence factor in *N. benthamiana* and that Roq1 is involved in the recognition of RipB.

## Introduction

Phytopathogenic bacteria have evolved a number of mechanisms to successfully infect host plants. The pathogenicity of many Gram‐negative bacteria depends on the Hrp type III secretion system that injects bacterial virulence proteins, called effector proteins, into plant cells (Galán *et al.*, 2014). The type III effectors manipulate host cellular processes, such as cytoskeletal rearrangements, transcriptional regulation, protein degradation and membrane trafficking, to suppress plant immunity and secure nutrients, resulting in an environment that allows the efficient growth of pathogens (Büttner, 2016).

To fight infection by pathogens, plants have evolved disease resistance (R) proteins to detect pathogen effectors inside plant cells. The recognition of effectors by R proteins induces strong defence responses, called effector‐triggered immunity (ETI), which include ion fluxes, production of reactive oxygen species (ROS), phytohormone accumulation and transcriptional activation of defence‐related genes (Cui *et al.*, 2015; Feys and Parker, 2000). The induction of the ETI response results in a hypersensitive response (HR), which is often accompanied by a rapid and programmed cell death, and restricts bacterial multiplication at the infection site. The pathogen effectors that are recognized by cognate plant R proteins are called avirulence (Avr) proteins, which restrict virulence to specific plants and often determine the host‐range specificity of a pathogen.

The largest class of R proteins is the nucleotide‐binding leucine‐rich repeat (NLR) proteins containing a nucleotide‐binding site (NBS) and C‐terminal leucine‐rich repeats (LRRs) (Cui *et al.*, 2015; Martin *et al.*, 2003). The NLR proteins are classified into two types, the Toll‐like interleukin‐1 receptor (TIR) type and the coiled coil (CC) type on the basis of the difference in the N‐terminal region. In many plant species, SGT1, a component of the HSP90‐SGT1‐RAR1 complex that acts as a molecular chaperone for NLR proteins, is required for the recognition of Avr effectors by NLR proteins (Peart *et al.*, 2002; Shirasu, 2009).

*Ralstonia solanacearum* is a Gram‐negative bacterium that causes bacterial wilt in more than 200 plant species belonging to over 50 botanical families (Hayward, 1991). The pathogen injects approximately 70 effector proteins into host cells via the Hrp type III secretion system during infection (Mukaihara *et al.*, 2010; Peeters *et al.*, 2013). To date, several effector proteins of *R. solanacearum* have been shown to act as avirulence determinants in certain plants. RipAA (formerly AvrA) triggers HR cell death and induces bacterial wilt resistance in *Nicotiana tabacum* (Carney and Denny, 1990). RipP1 (formerly PopP1), an acetyltransferase effector of the YopJ family, restricts bacterial growth in *Petunia* St40 (Lavie *et al.*, 2002). RipP2 (formerly PopP2), another member of the YopJ family, induces bacterial wilt resistance in *Arabidopsis thaliana* Nd‐1 (Deslandes *et al.*, 2003). RipAX2 (formerly Rip36), a putative Zn‐dependent protease effector, restricts the growth of *R. solanacearum* in the eggplant wild relative *Solanum torvum* (Nahar *et al.*, 2014) and eggplant AG91‐25 (Morel *et al.*, 2018).

The two type III effectors RipP1 and RipAA from the *R. solanacearum* strain GMI1000 have been shown to act as avirulence determinants in *N. tabacum* and *N. benthamiana* because the *ripP1 ripAA* mutant of GMI1000 becomes fully virulent on both plants (Poueymiro *et al.*, 2009). However, there are no obvious relationships between the ability to induce HR cell death in *N. tabacum* and the presence of *ripP1* and *ripAA* in the genome of 22 Japanese *R. solanacearum* strains (Liu *et al.*, 2009). In contrast to GMI1000, all of the *ripP1 ripAA* mutants of four representative Japanese tobacco‐avirulent strains caused no wilting symptoms in *N. tabacum*, whereas they induced delayed HR on leaves compared with the wild‐type (WT) strains (Chen *et al.*, 2018). Therefore, it has been postulated that many Japanese *R. solanacearum* strains possess as‐yet‐unidentified avirulence factors other than RipP1 and RipAA.

Our previous study showed that RipP1 and RipAA trigger ROS bursts before the development of HR cell death in *N. benthamiana* leaves (Nakano *et al.*, 2015). ROS bursts are strongly induced in an early event of recognition of avirulence factors from bacteria, fungi, oomycetes and viruses (Adachi *et al.*, 2015; Doke and Ohashi, 1988; Levine *et al.*, 1994; Vera‐Estrella *et al.*, 1992), indicating that a strong ROS burst can be used as a good indicator for the screening of Avr effectors. In this study, we screened *R. solanacearum* effectors for their ability to induce a strong ROS burst in *N. benthamiana* and identified a novel Avr effector from the Japanese *R. solanacearum* strain RS1000. We provide evidence that RipB is a major Avr effector that restricts the host range of RS1000 to *N. benthamiana* and several *Nicotiana* species. We also examined the cognate R protein involved in the recognition of RipB in *N. benthamiana*.

## Results

### RipB induces ETI in *N. benthamiana*

**Figure 1:**
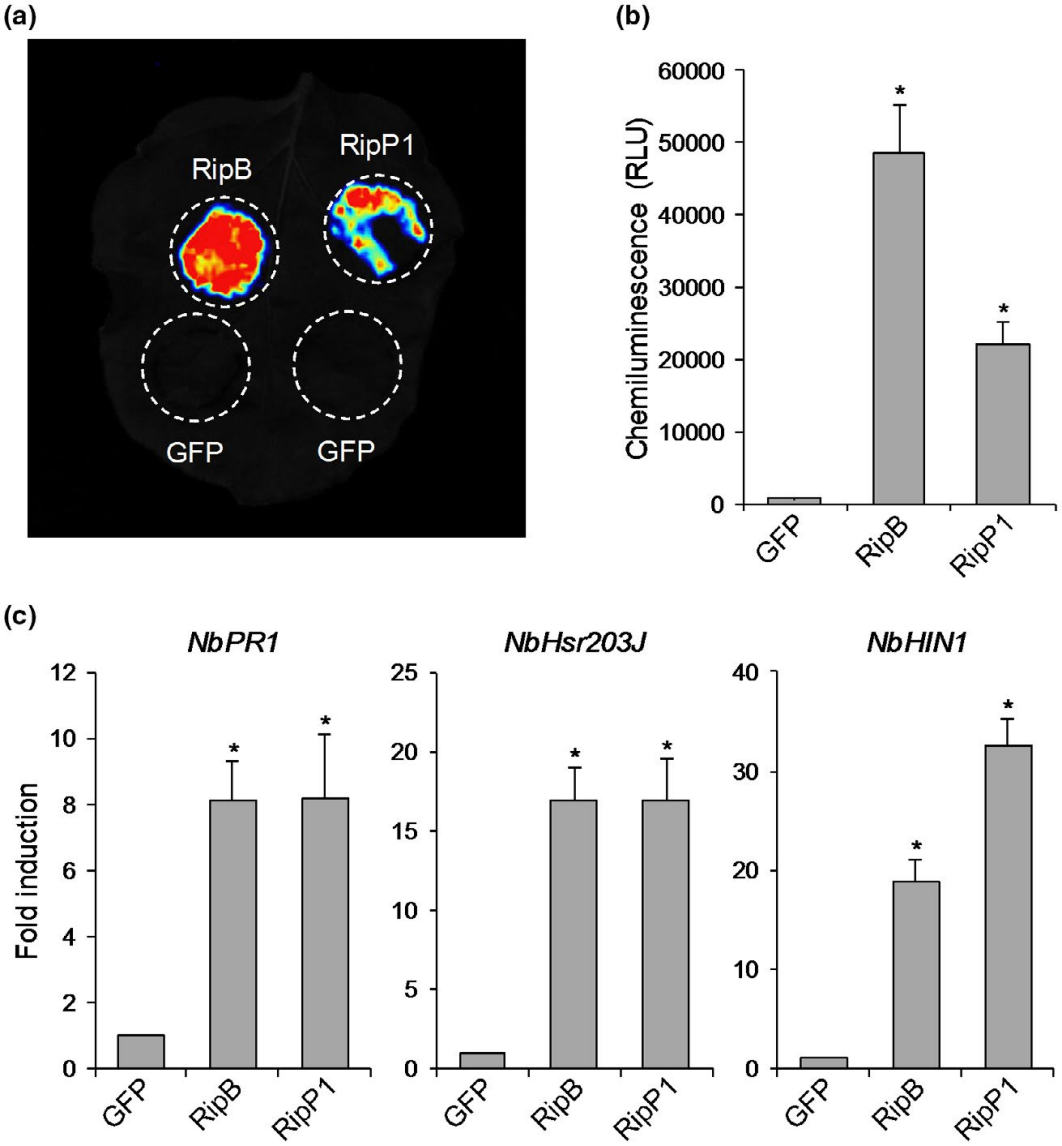
Effect of RipB expression on effector‐triggered immunity in *Nicotiana benthamiana*. Leaves were infiltrated with *Agrobacterium tumefaciens* harbouring the binary vector expressing RipB, RipP1 or the GFP. (a) ROS bursts in leaves expressing RipB, RipP1 and GFP. Reactive oxygen species (ROS) production was measured 18 h after agroinfiltration and detected as photon counts using a chemiluminescence probe. (b) Total photon counts for 2 min in (a). Values are means ± SD of six replicates. Asterisks denote statistically significant differences compared with the GFP control (*P* < 0.01, Student’s *t*‐test). (c) Expression levels of defence‐related genes in leaves expressing RipB and RipP1. Total RNA was isolated from leaves 1 day after agroinfiltration. Expression levels were determined by real‐time PCR analysis and normalized to that in the GFP control. Values are means ± SD of three replicates. Asterisks denote statistically significant differences compared with the GFP control (*P* < 0.01, Student’s *t*‐test). All experiments were repeated three times with similar results and representative results are shown.

We previously identified 68 effector proteins from the tobacco‐avirulent *R. solanacearum* strain RS1000 (Mukaihara *et al.*, 2010) and cloned the coding region of each effector gene into a binary vector under the control of the constitutive promoter (Nahar *et al.*, 2014). To identify as‐yet‐unidentified Avr effectors of Japanese *R. solanacearum* strains, we transiently expressed RS1000 effector proteins in *N. benthamiana* leaves by agroinfiltration and examined their ability to induce a strong ROS burst in the infiltrated area. In this screening, we observed that the expression of RipB induced a strong ROS burst in *N. benthamiana* leaves (Fig. 1a). The level of ROS production induced by RipB was approximately two‐fold higher than that by RipP1, an already‐known Avr effector that induces HR cell death in *N. benthamiana* (Poueymiro *et al.*, 2009) (Fig. 1b). We found no effector proteins, except for RipB, that induced a stronger ROS burst than RipP1 (data not shown). The expression of RipB did not induce HR cell death in *N. benthamiana* leaves and only induced leaf chlorosis 7 days after agroinfiltration, whereas the expression of RipP1 induced a typical HR cell death in the infiltrated area 4 days after agroinfiltration (Fig. S1).

To evaluate the effect of RipB expression on plant ETI responses, we monitored the expressions of several defence‐related genes in *N. benthamiana*. As expected, the expression of RipB, as well as RipP1, induced the expressions of the salicylic acid (SA) signalling marker gene *NbPR1* (Ward *et al.*, 1991) and the HR marker genes *NbHsr203J* and *NbHIN1* (Gopalan *et al.*, 1996; Pontier *et al.*, 1994) in leaves compared with the GFP control 1 day after agroinfiltration (Fig. 1c). These findings indicate that RipB has the ability to induce ETI responses in *N. benthamiana*.

### RipB‐induced ETI is dependent on SGT1 and ICS1

**Figure 2:**
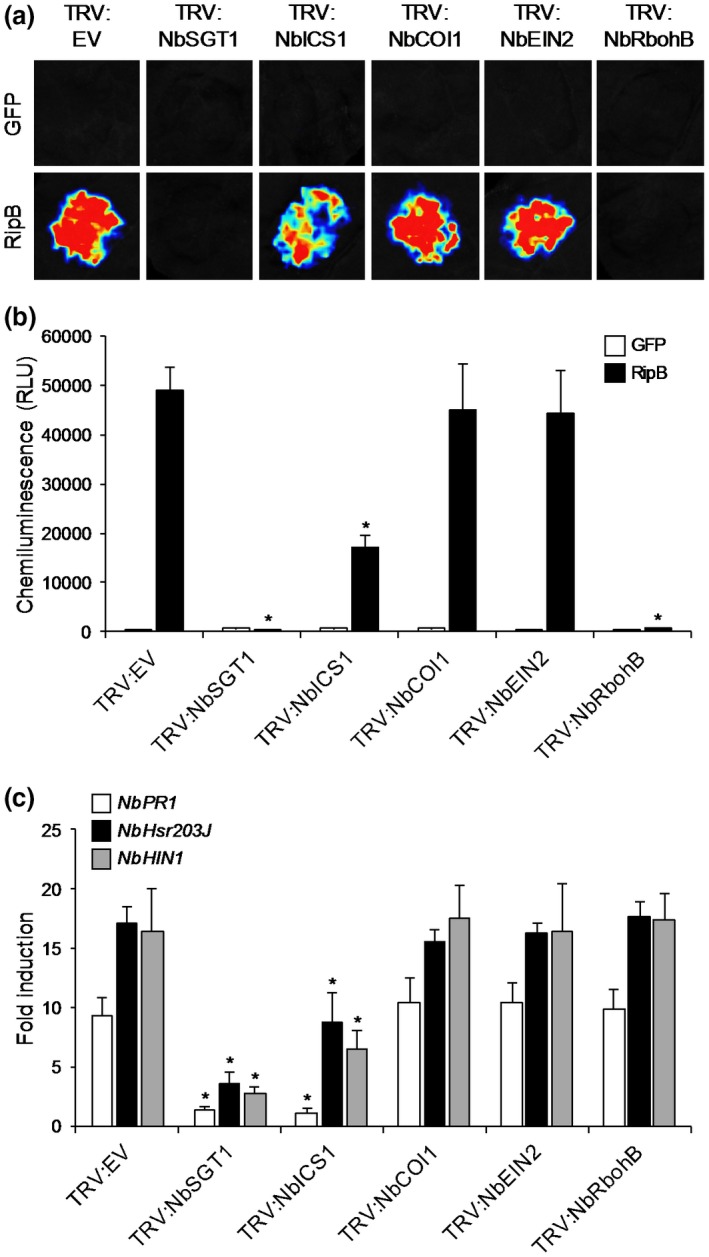
Effects of *NbSGT1*, *NbICS1*, *NbCOI1*, *NbEIN2* and *NbRbohB* silencing on effector‐triggered immunity in *Nicotiana benthamiana*. Seedlings of *N. benthamiana* were coinfiltrated with *Agrobacterium tumefaciens* harbouring pTRV1 and that harbouring pTRV2 (TRV:EV), pTRV2:NbSGT1 (TRV:NbSGT1), pTRV2:NbICS1 (TRV:NbICS1), pTRV2:NbCOI1 (TRV:NbCOI1), pTRV2:NbEIN2 (TRV:NbEIN2) or pTRV2:NbRbohB (TRV:NbRbohB). Leaves of silenced plants were infiltrated with *A. tumefaciens* harbouring the binary vector expressing RipB or the GFP. (a) Reactive oxygen species (ROS) bursts in leaves expressing RipB and GFP. ROS production was measured 18 h after agroinfiltration and detected as photon counts using a chemiluminescence probe. (b) Total photon counts for 2 min in (a). Values are means ± SD of six replicates. Asterisks denote statistically significant differences compared with the TRV:EV control (*P* < 0.01, Student’s *t*‐test). (c) Expression levels of defence‐related genes in leaves of the control and gene‐silenced plants expressing RipB. Total RNA was isolated from leaves 1 day after agroinfiltration. Expression levels were determined by real‐time PCR analysis and normalized to that in the GFP control. Values are means ± SD of three replicates. Asterisks denote statistically significant differences compared with the TRV:EV control (*P* < 0.01, Student’s *t*‐test). All experiments were repeated three times with similar results and representative results are shown.

In many plant species, the SGT1 component of the HSP90‐SGT1‐RAR1 molecular chaperone complex is required for the function of NLR proteins (Shirasu, 2009). To determine whether SGT1 is involved in the ETI responses induced by RipB, we silenced the expression of *NbSGT1* in *N. benthamiana* using a virus‐induced gene silencing (VIGS) system (Fig. S2). The RipB‐induced ROS burst completely disappeared in the *NbSGT1*‐silenced plants as well as the plants with silenced *NbRbohB* (Fig. 2a,b), which encodes the ROS‐producing enzyme (Yoshioka *et al.*, 2003). The RipB‐induced expression levels of defence‐related genes significantly decreased in the *NbSGT1*‐silenced plants compared with the control plants (Fig. 2c), indicating that NbSGT1 is required for the ETI responses induced by RipB. These findings suggest that RipB is recognized by an unidentified NLR protein in *N. benthamiana.*

The recognition of Avr effector proteins by cognate NLR proteins activates a wide array of signalling pathways in plant cells (Cui *et al.*, 2015; Feys and Parker, 2000). To identify the signalling pathway acting downstream of RipB recognition, we silenced the expressions of *ICS1, COI1* and *EIN2,* which respectively regulate SA, jasmonic acid (JA) and ethylene (ET) hormonal signalling pathways, which play important roles in plant immunity (Pieterse *et al.*, 2012), in *N. benthamiana* using the VIGS system (Fig. S2). The RipB‐induced ROS burst and the expressions of defence‐related genes were partially reduced in the *NbICS1*‐silenced plants, but not in the *NbCOI1*‐ and *NbEIN2*‐silenced plants, compared with the control plants (Fig. 2). These findings indicate that SA signalling, but not JA and ET signalling, is involved in the ETI responses induced by RipB.

### RipB acts as a major Avr effector that determines bacterial pathogenicity in *N. benthamiana*

**Figure 3:**
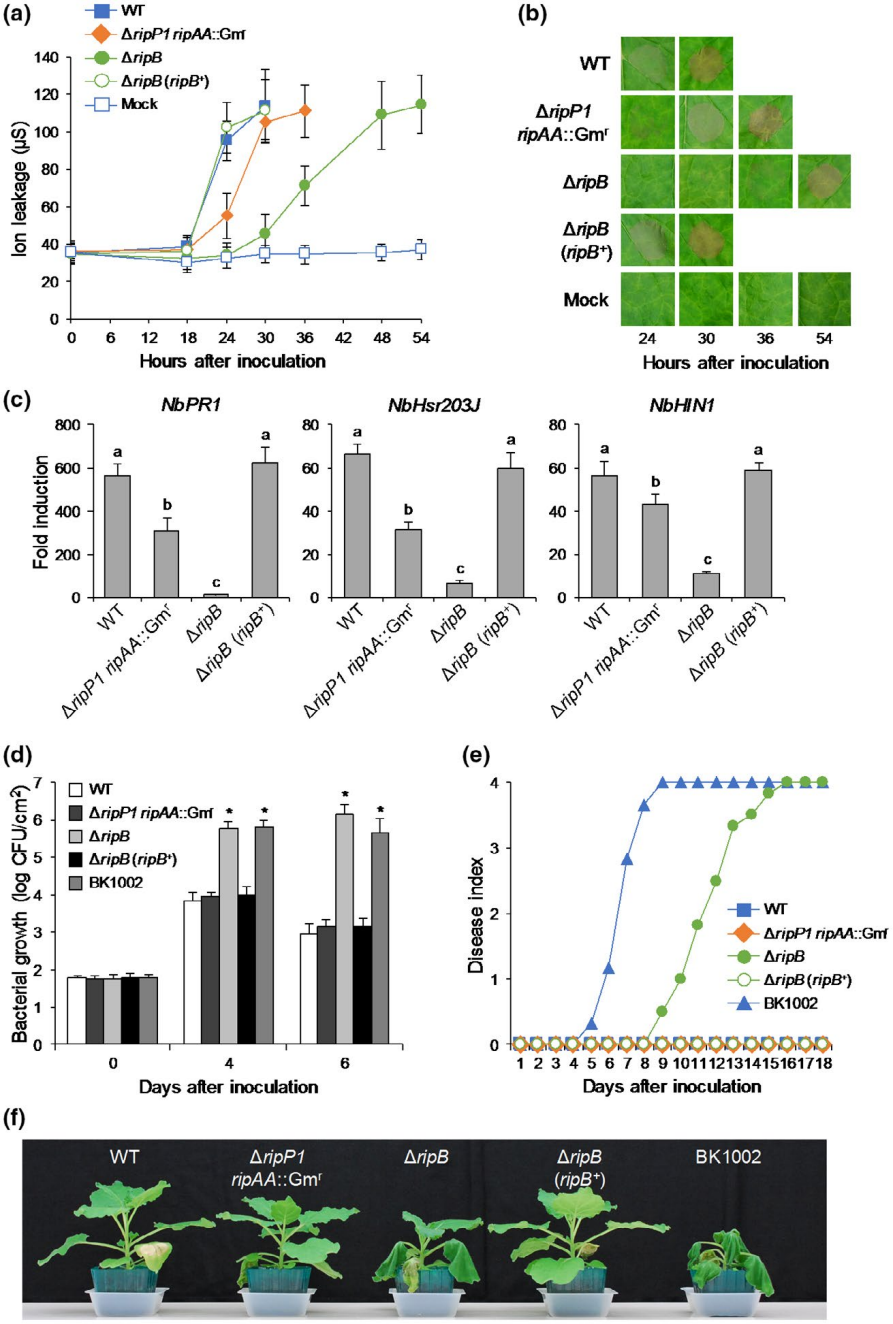
Hypersensitive response (HR) and virulence tests of *Nicotiana benthamiana*. Leaves were infiltrated with the suspensions of *Ralstonia solanacearum* RS1002 (WT) and the Δ*ripP1 ripAA*::Gm^r^, Δ*ripB* and Δ*ripB* (*ripB^+^*) mutants, tobacco‐virulent strain BK1002 and water (mock). (a) HR in leaves inoculated with *R. solanacearum* mutants. The degree of ion leakage from leaves was measured at the indicated times after inoculation. Values are means ± SD of six replicates. (b) HR development in leaves in (a). Photographs were taken at the indicated times after inoculation. (c) Expression levels of defence‐related genes in leaves inoculated with *R. solanacearum* mutants. Total RNA was isolated from leaves 12 h after inoculation. Expression levels were determined by real‐time PCR analysis and normalized to that in the mock treatment. Values are means ± SD of three replicates. Different letters denote statistically significant differences (*P* < 0.05, one‐way ANOVA with Tukey–Kramer HSD test). (d) Growth of *R. solanacearum* mutants in leaves. The bacterial population was determined on the indicated days after inoculation. Values are means ± SD of four replicates. Asterisks denote statistically significant differences compared with the WT strain (*P* < 0.01, Student’s *t*‐test). (e) Disease development in plants inoculated with *R. solanacearum* mutants. The severity of wilting symptoms was scored daily using the disease index. (f) Disease symptoms in plants in (e). Photographs were taken 11 days after inoculation. All experiments were repeated three times with similar results and representative results are shown.

To examine the role of RipB in the *R. solanacearum*–*N. benthamiana* interaction, we generated a Δ*ripB* mutant of the *R. solanacearum* strain RS1002, a nalixidic acid‐resistant (Nal^r^) derivative of RS1000. Because RipP1 and RipAA of the *R. solanacearum* strain GMI1000 have been shown to act as Avr effectors in *N. benthamiana* (Poueymiro *et al.*, 2009), we also generated a Δ*ripP1 ripAA*::Gm^r^ mutant of RS1002. When the WT RS1002 was infiltrated into the leaves of *N. benthamiana*, an HR cell death accompanied by electrolyte leakage was observed 24 h after inoculation in the infiltrated area and was fully developed 30 h after inoculation (Fig. 3a,b). The Δ*ripP1 ripAA*::Gm^r^ mutant showed a slightly delayed development of HR in the inoculated leaves compared with the WT strain. We observed that the Δ*ripB* mutant induced a less severe HR in the inoculated leaves than the Δ*ripP1 ripAA*::Gm^r^ mutant. The introduction of *ripB* ^+^ into the Δ*ripB* mutant completely recovered the delayed development of HR in the infiltrated area to the WT level. Next, we monitored the expressions of defence‐related genes in *N. benthamiana* leaves inoculated with the *R. solanacearum* mutants. The WT strain significantly induced the expressions of the SA signalling and HR marker genes in the inoculated leaves 12 h after inoculation, whereas the Δ*ripP1 ripAA*::Gm^r^ mutant induced their expressions at almost half levels compared with the WT strain (Fig. 3c). Notably, the Δ*ripB* mutant induced markedly reduced expressions of defence‐related genes in the inoculated leaves compared with the WT strain. The introduction of *ripB* ^+^ completely recovered the expression levels of the marker genes to the WT level. These findings indicate that RipB induces ETI responses during the infection of *R. solanacearum* in *N. benthamiana*.

To investigate whether RipB acts as an Avr protein in *N. benthamiana*, we monitored the growth of the *R. solanacearum* mutants and the development of wilting symptoms in plants. The bacterial growth of the Δ*ripP1 ripAA*::Gm^r^ mutant showed no difference compared with that of the WT strain in the inoculated leaves 4 and 6 days after inoculation (Fig. 3d). The inoculation of the Δ*ripP1 ripAA*::Gm^r^ mutant of RS1002 caused no wilting symptoms in *N. benthamiana* (Fig. 3e,f). On the other hand, the bacterial growth of the Δ*ripB* mutant was 100‐fold higher than that of the WT strain, which is nearly equal to that of the tobacco‐virulent strain BK1002 (Fig. 3d). Notably, the inoculation of the Δ*ripB* mutant caused wilting symptoms in *N. benthamiana* (Fig. 3e,f). However, the disease development was much slower than that caused by BK1002. The introduction of *ripB^+^* into the Δ*ripB* mutant inhibited the mutant’s growth and the disease development in *N. benthamiana* (Fig. 3d–f). We also performed the virulence test using the stem inoculation method and obtained similar results (Fig. S3). Collectively, these observations indicate that RipB, but neither RipP1 nor RipAA, acts as a major Avr effector that restricts the host range of RS1002 to *N. benthamiana*.

### RipP1 and RipAA act as minor Avr effectors in *N. benthamiana*

**Figure 4:**
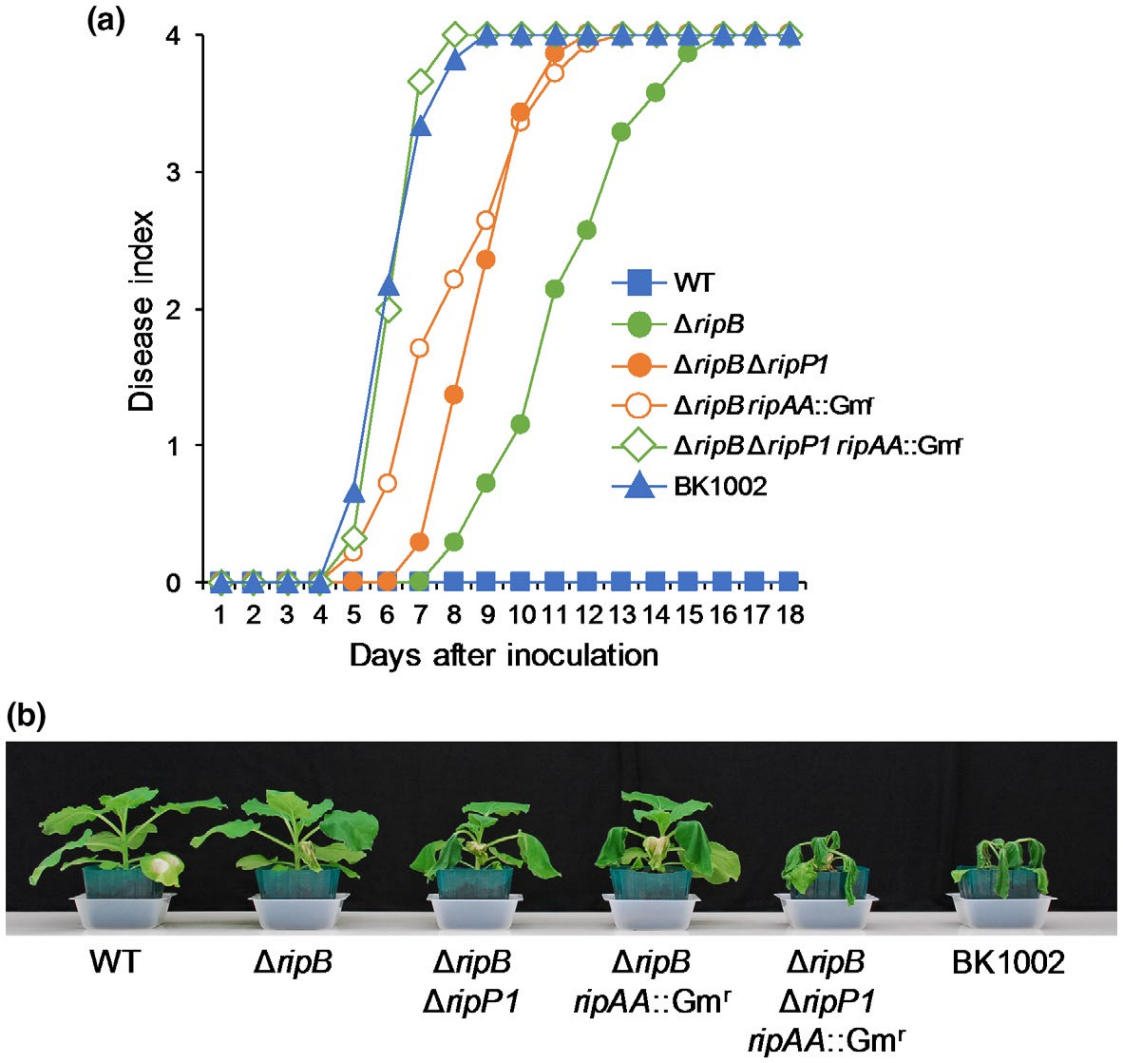
Virulence of *Ralstonia solanacearum* mutants in *Nicotiana benthamiana*. Leaves were inoculated with the suspensions of *R. solanacearum* RS1002 (WT) and the Δ*ripB*, Δ*ripB ripAA*::Gm^r^, Δ*ripB* Δ*ripP1* and Δ*ripB* Δ*ripP1 ripAA*::Gm^r^ mutants, and tobacco‐virulent strain BK1002. (a) Disease development in plants inoculated with *R. solanacearum* mutants. The severity of wilting symptoms was scored daily using the disease index. (b) Disease symptoms in plants in (a). Photographs were taken 9 days after inoculation. All experiments were repeated three times with similar results and representative results are shown.

Although the Δ*ripB* mutant was able to grow and cause wilting symptoms in *N. benthamiana*, it induced a much slower disease development than BK1002 (Fig. 3e,f). Because the Δ*ripP1 ripAA*::Gm^r^ mutant showed a reduced induction of ETI responses to *R. solanacearum* in *N. benthamiana* leaves (Fig. 3a–c), we hypothesized that the presence of RipP1 and/or RipAA affects the virulence of the Δ*ripB* mutant in *N. benthamiana*. To verify this hypothesis, we generated Δ*ripB ripAA*::Gm^r^ and Δ*ripB* Δ*ripP1* double mutants and a Δ*ripB* Δ*ripP1 ripAA*::Gm^r^ triple mutant of RS1002, and examined their virulence in *N. benthamiana*. We observed that both Δ*ripB ripAA*::Gm^r^ and Δ*ripB* Δ*ripP1* mutants caused more severe wilting symptoms in *N. benthamiana* than the Δ*ripB* mutant, whereas the disease development caused by these mutants was still slower than that caused by BK1002 (Fig. 4a,b). Notably, the Δ*ripB* Δ*ripP1 ripAA*::Gm^r^ mutant caused very severe wilting symptoms in *N. benthamiana* nearly equal to those caused by BK1002. These observations indicate that RipP1 and RipAA act as minor Avr effectors that reduce bacterial virulence in *N. benthamiana*.

### RipB_BK_ isolated from virulent BK1002 loses the ability to induce ETI in *N. benthamiana*

**Figure 5:**
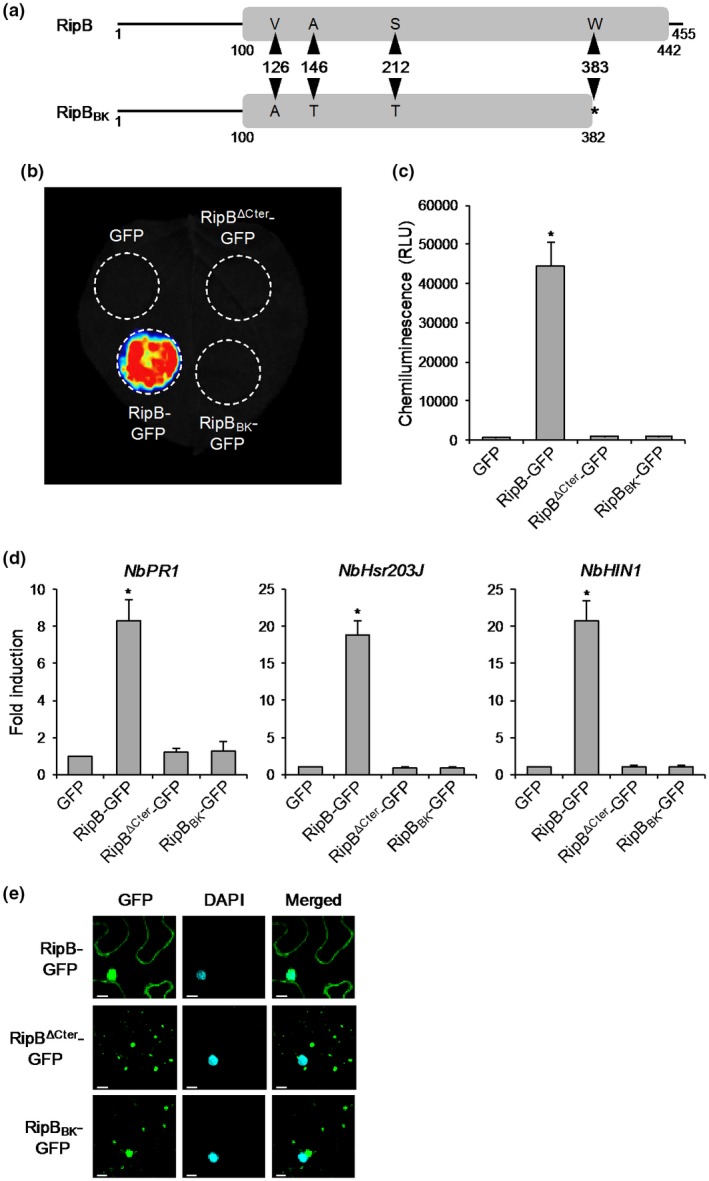
Functional analyses of RipB_BK_ from tobacco‐virulent *Ralstonia solanacearum* BK1002. (a) Schematic diagram of domain structure of RipB from tobacco‐avirulent RS1000 and RipB_BK_ from tobacco‐virulent BK1002. The positions of amino acid residues are indicated by numbers. Black arrowheads denote different amino acid residues between RipB and RipB_BK_. The nonsense mutation in RipB_BK_ is indicated by an asterisk. The nucleoside hydrolase domain is indicated as a grey box. (b) ROS bursts in leaves expressing RipB‐GFP fusions. Leaves were infiltrated with *Agrobacterium tumefaciens* harbouring the binary vector expressing GFP‐tagged RipB, RipB^ΔCter^ or RipB_BK_, or GFP alone. Reactive oxygen species (ROS) production was measured 18 h after agroinfiltration and detected as photon counts using a chemiluminescence probe. (c) Total photon counts for 2 min in (b). Values are means ± SD of six replicates. The asterisk denotes a statistically significant difference compared with the GFP control (*P* < 0.01, Student’s *t*‐test). (d) Expression levels of defence‐related genes in leaves expressing RipB‐GFP fusions. Total RNA was isolated from leaves 1 day after agroinfiltration. Expression levels were determined by real‐time PCR analysis and normalized to that in the GFP control. Values are means ± SD of three replicates. Asterisks denote statistically significant differences compared with the GFP control (*P* < 0.01, Student’s *t*‐test). (e) Subcellular localization of RipB‐GFP, RipB^ΔCter^‐GFP and RipB_BK_‐GFP in plant cells. Fluorescence signals were observed 1 day after agroinfiltration by confocal microscopy. Bars, 10 μm. All experiments were repeated three times with similar results and representative results are shown.

RipB is one of the core effectors that are conserved among the effector repertoires in the *R. solanacearum* species complex (Peeters *et al.*, 2013). From whole‐genome sequencing analysis of *R. solanacearum* BK1002, we found that this strain also possesses *ripB* on its chromosome (data not shown). We compared the amino acid sequence of RipB from BK1002 (RipB_BK_) with that of RipB from RS1000 (RipB) and found that RipB_BK_ contains three nonsynonymous substitutions at positions 126, 146 and 212, and a nonsense mutation at position 383 that causes a deletion of 73 C‐terminal amino acids corresponding to positions 383 to 455 of full‐length RipB (Fig. 5a). The close homologues of RipB, such as HopQ1 and XopQ, have been identified from *Pseudomonas* and *Xanthomonas* strains, respectively (Schultink *et al.*, 2017). The three amino acids substituted in RipB_BK_ were variable and not conserved among the eight RipB homologues from *R. solanacearum*, *Pseudomonas* and *Xanthomonas* strains (Fig. S4). On the other hand, the tryptophan residue at position 383 is conserved among the RipB homologues, and the C‐terminal deletion was only observed in RipB_BK_.

Because *R. solanacearum* BK1002 showed high virulence in *N. benthamiana* (Figs. 3d–f and 4), we tested whether RipB_BK_ can elicit ETI responses in this plant. First, we transiently expressed the C‐terminal green fluorescent protein (GFP)‐tagged RipB from RS1000 (RipB‐GFP) in *N. benthamiana* leaves by agroinfiltration (Fig. S5a). The expression of RipB‐GFP, but not that of GFP alone, triggered a strong ROS burst nearly equal to that triggered by RipB in the infiltrated area (Fig. 5b,c), indicating that the GFP tag has little effect on the function of RipB. As expected, the expression of the C‐terminal GFP‐tagged RipB from BK1002 (RipB_BK_‐GFP) induced no ROS burst in *N. benthamiana* leaves. We constructed RipB^ΔCter^, in which the amino acid positions from 383 to 455 of RipB from RS1000 were deleted, and confirmed that the deletion of the 73 C‐terminal amino acids completely abolished the ability of RipB to induce ROS bursts. The expressions of the defence‐related genes were induced by the expression of RipB‐GFP, but not those of RipB_BK_‐GFP, RipB^ΔCter^‐GFP and GFP alone (Fig. 5d). The prolonged expression of RipB‐GFP, but not those of RipB^ΔCter^‐GFP, RipB_BK_‐GFP and GFP alone, induced leaf chlorosis 7 days after agroinfiltration (Fig. S5b). These findings clearly showed that RipB_BK_ loses the ability to induce ETI responses in *N. benthamiana* owing to the C‐terminal deletion.

To clarify the subcellular localization of RipB in plant cells, we expressed the RipB‐GFP, RipB^ΔCter^‐GFP and RipB_BK_‐GFP proteins in *N. benthamiana* leaves. Confocal laser scanning microscopy revealed that RipB‐GFP localized to both the cytoplasm and nucleus 1 day after agroinfiltration (Fig. 5e). On the other hand, the fluorescence signals of RipB^ΔCter^‐GFP and RipB_BK_‐GFP localized to punctate structures of 1–10 μm size range within the plant cell. The GFP signal did not merge with that of 4′,6‐diamidino‐2‐phenylindole (DAPI), a fluorescent stain for the nucleus, indicating that neither RipB^ΔCter^‐GFP nor RipB_BK_‐GFP localized to the nucleus. This finding indicates that the deletion of the 73 C‐terminal amino acids affects the nucleocytoplasmic localization of RipB in plant cells.

**Figure 6:**
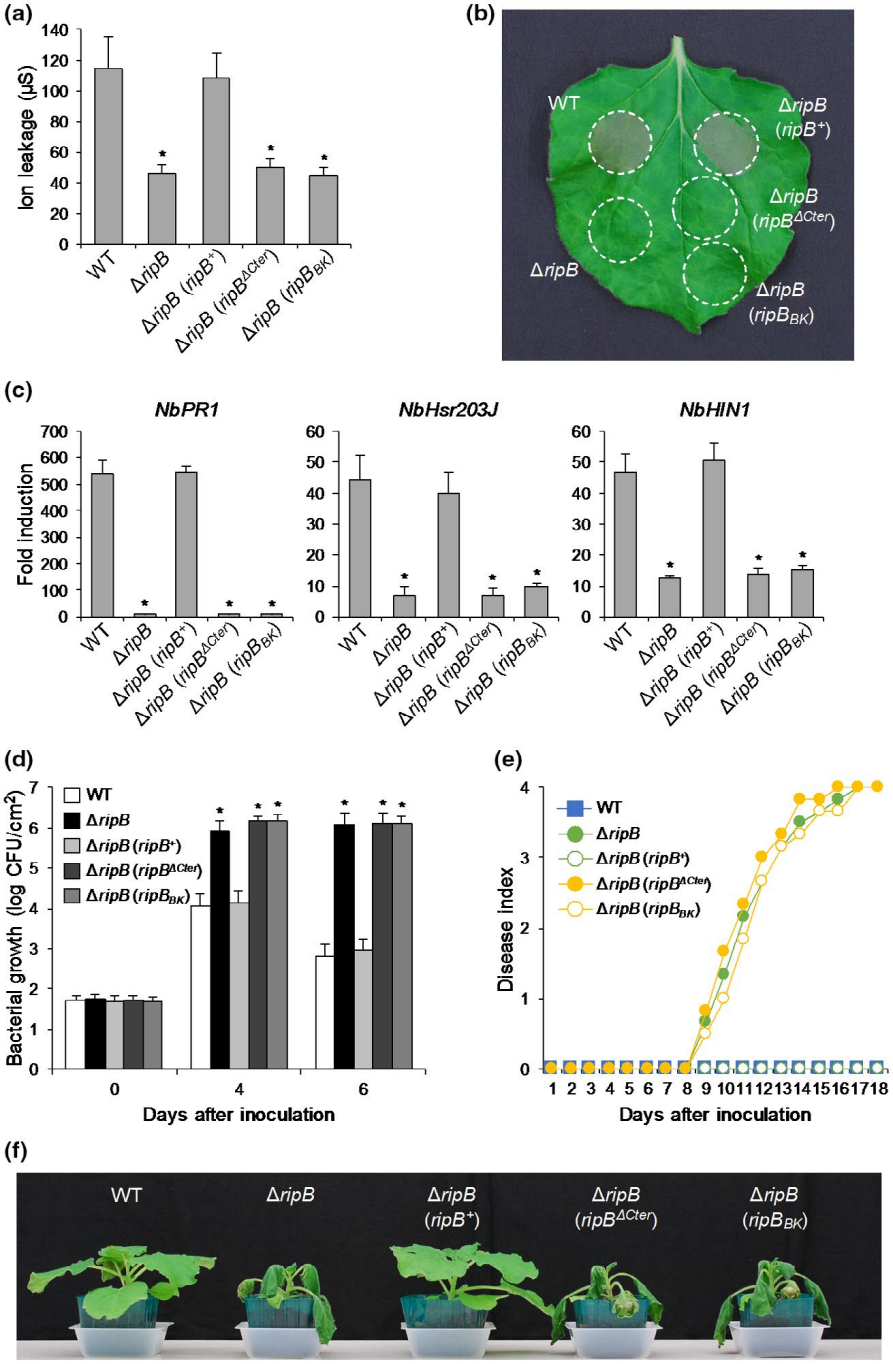
Effect of the C‐terminal deletion on the recognition of RipB in *Nicotiana benthamiana*. Leaves were inoculated with the suspensions of *Ralstonia solanacearum* RS1002 (WT), and the Δ*ripB*, Δ*ripB* (*ripB^+^*), Δ*ripB* (*ripB^ΔCter^*) and Δ*ripB* (*ripB_BK_*) mutants. (a) Hypersensitive reposnse (HR) in leaves inoculated with *R. solanacearum* mutants. The degree of ion leakage from leaves was measured 24 h after inoculation using a conductivity meter. Values are means ± SD of six replicates. Asterisks denote statistically significant differences compared with the WT strain (*P* < 0.01, Student’s *t*‐test). (b) HR development in leaves in (a). Photographs were taken 24 h after inoculation. (c) Expression levels of defence‐related genes in leaves inoculated with *R. solanacearum* mutants. Total RNA was isolated from leaves 12 h after inoculation. Expression levels were determined by real‐time PCR analysis and normalized to that in the mock treatment. Values are means ± SD of three replicates. Asterisks denote statistically significant differences compared with the WT strain (*P* < 0.01, Student’s *t*‐test). (d) Growth of *R. solanacearum* mutants in leaves. The bacterial population was determined on the indicated days after inoculation. Values are means ± SD of four replicates. Asterisks denote statistically significant differences compared with the WT strain (*P* < 0.01, Student’s *t*‐test). (e) Disease development in plants inoculated with *R. solanacearum* mutants. The severity of wilting symptoms was scored daily using the disease index. (f) Disease symptoms in plants in (e). Photographs were taken 15 days after inoculation. All experiments were repeated three times with similar results and representative results are shown.

To test whether the C‐terminal‐truncated RipB mutants are recognized by *N. benthamiana* during their infection, we introduced *ripB^ΔCter^* and *ripB_BK_* into the Δ*ripB* mutant. As expected, neither *ripB^ΔCter^* nor *ripB_BK_* recovered the deficiencies of the Δ*ripB* mutant in the induction of HR cell death (Fig. 6a,b) and defence‐related gene expressions (Fig. 6c) in *N. benthamiana*. Moreover, the Δ*ripB* mutants introduced using *ripB^ΔCter^* and *ripB_BK_* showed no attenuation of bacterial growth (Fig. 6d) or disease development (Fig. 6e,f) in *N. benthamiana* compared with the parental strain. These findings clearly show that the C‐terminal‐truncated RipB_BK_ does not act as an Avr effector in *N. benthamiana*.

### Difference in RipB recognition among *Nicotiana* plants

**Figure 7:**
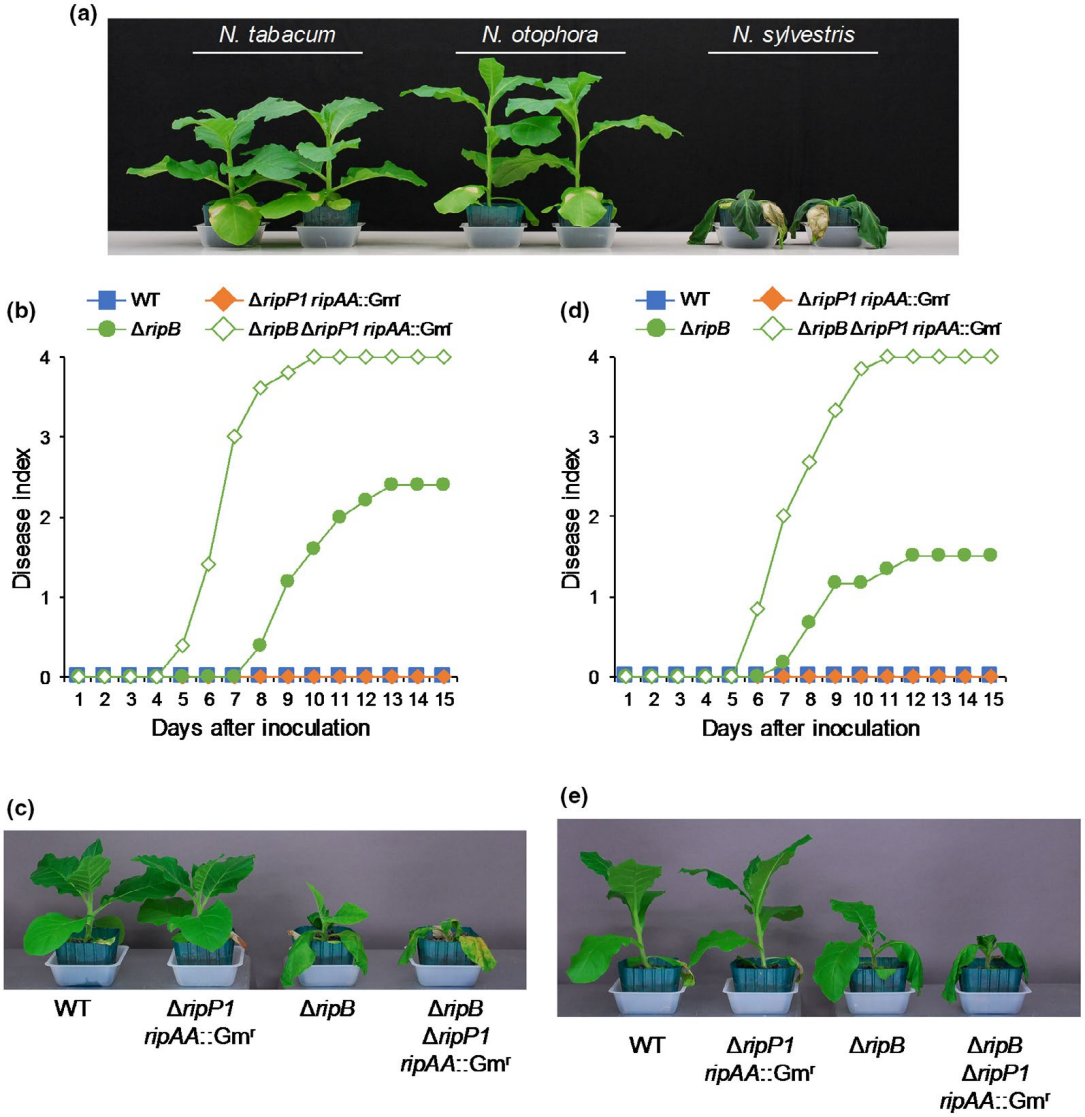
Pathogenicity of *Ralstonia solanacearum* mutants in *Nicotiana* plants. Leaves were inoculated with the suspensions of *R. solanacearum* RS1002 (WT) and the Δ*ripP1 ripAA*::Gm^r^, Δ*ripB* and Δ*ripB* Δ*ripP1 ripAA*::Gm^r^ mutants. (a) Disease symptoms in *N. tabacum*, *N. otophora* and *N. sylvestris* inoculated with RS1002. Photographs were taken 9 days after inoculation. Disease development in *N. tabacum* (b) and *N. otophora* (d) inoculated with *R. solanacearum* mutants. The severity of wilting symptoms was scored daily using the disease index. Disease symptoms in *N. tabacum* (c) and *N. otophora* (e) inoculated with *R. solanacearum* mutants. Photographs were taken 9 days after inoculation. All experiments were repeated three times with similar results and representative results are shown.

To examine whether RipB is recognized by *Nicotiana* plants other than *N. benthamiana*, we evaluated the pathogenicity of RS1002 in several *Nicotiana* plants such as *N. tabacum*, *N. otophora* and *N. sylvestris*. When RS1002 was infiltrated into plant leaves, we observed severe wilting symptoms in *N. sylvestris*, but not in *N. tabacum* or *N. otophora*, 9 days after inoculation (Fig. 7a). This finding indicates that *N. tabacum* and *N. otophora* are resistant to RS1002. Similarly to *N. benthamiana*, although the Δ*ripP1 ripAA*::Gm^r^ double mutant of RS1002 caused no wilting symptoms, the Δ*ripB* mutant and the Δ*ripB* Δ*ripP1 ripAA*::Gm^r^ mutant, respectively, caused mild and severe wilting symptoms in *N. tabacum* (Fig. 7b,c) and *N. otophora* (Fig. 7d,e). The transient expression of RipB induced leaf chlorosis 7 days after agroinfiltration in both *N. tabacum* and *N. otophora*, but not in *N. sylvestris* (Fig. S6)*.* These observations suggest that RipB is recognized as an Avr effector in *N. tabacum* and *N. otophora*, but not in *N. sylvestris*.

### RipB is recognized by Roq1

**Figure 8:**
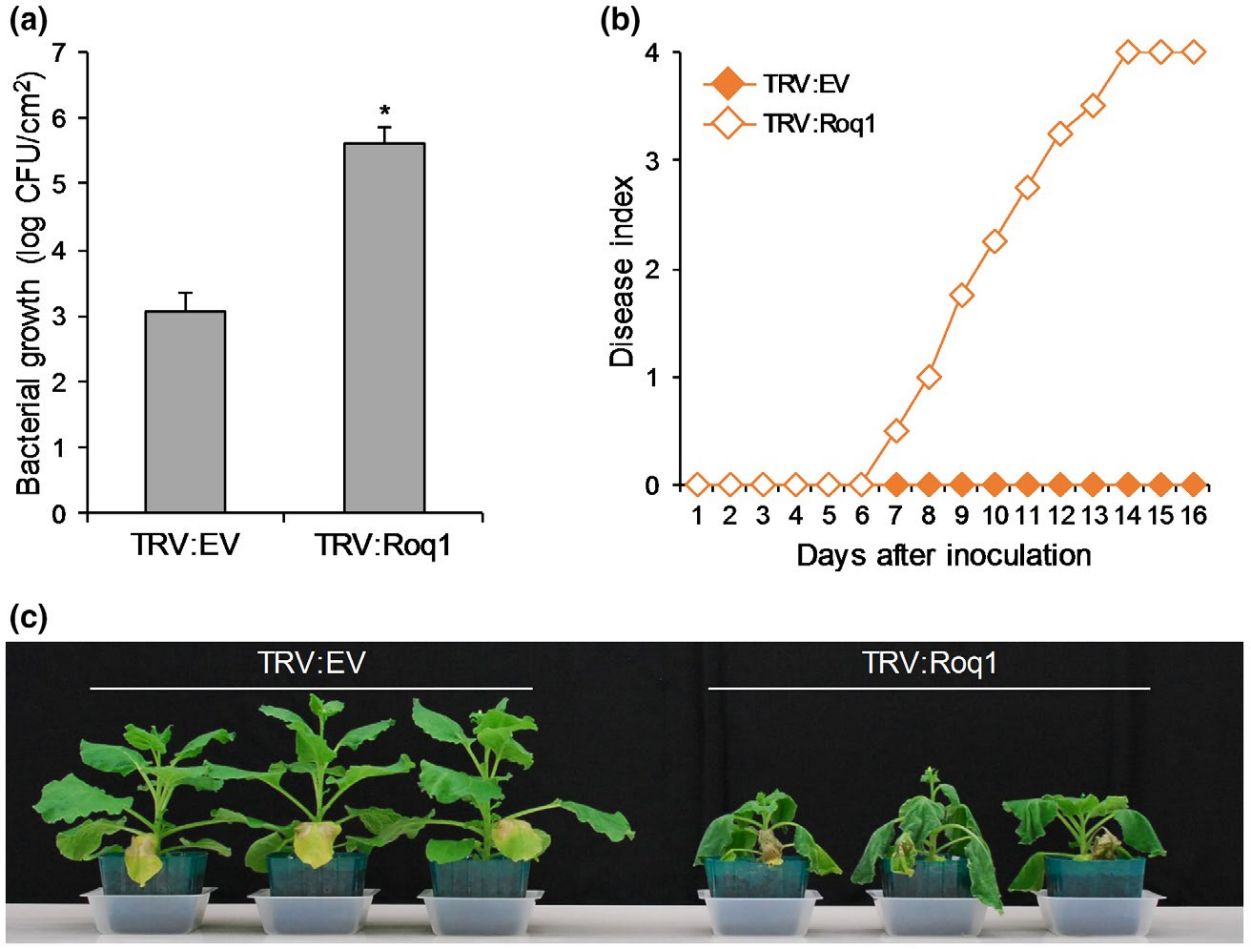
Effect of *Roq1* silencing on the resistance of *Nicotiana benthamiana* against *Ralstonia solanacearum*. Seedlings of *N. benthamiana* were coinfiltrated with *Agrobacterium tumefaciens* harbouring pTRV1 and that harbouring pTRV2 (TRV:EV) or pTRV2:Roq1 (TRV:Roq1). Leaves of silenced plants were inoculated with the suspension of the Δ*ripP1 ripAA*::Gm^r^ mutant of *R. solanacearum* RS1002. (a) Growth of *R. solanacearum* in the control and *Roq1*‐silenced plants. The bacterial population was determined 6 days after inoculation. Values are means ± SD of four replicates. The asterisk denotes a statistically significant difference compared with the TRV:EV control (*P* < 0.01, Student’s *t*‐test). (b) Disease development in the control and *Roq1*‐silenced plants inoculated with *R. solanacearum*. The severity of wilting symptoms was scored daily using the disease index. (c) Disease symptoms in the control and *Roq1*‐silenced plants in (b). Photographs were taken 10 days after inoculation. All experiments were repeated three times with similar results and representative results are shown.

Recently, Schultink *et al. *(2017) have reported that the *N. benthamiana* NLR protein Roq1 recognizes the *Xanthomonas* effector XopQ, which is a close homologue of RipB. The intact orthologue gene *Roq1* is present in *N. tabacum*, but not in *N. sylvestris*. Because RipB induced bacterial wilt resistance in *N. tabacum*, but not in *N. sylvestris* (Figs 7a–c and S6a,c), we hypothesized that Roq1 is involved in the recognition of RipB. To verify this hypothesis, we silenced the expression of *Roq1* in *N. benthamiana* using the VIGS system (Fig. S7) and inoculated the Δ*ripP1 ripAA*::Gm^r^ mutant, which lacks Avr effectors other than RipB. The bacterial growth of the Δ*ripP1 ripAA*::Gm^r^ mutant increased 100‐fold in the *Roq1*‐silenced plants compared with the control plants 6 days after inoculation (Fig. 8a). The Δ*ripP1 ripAA*::Gm^r^ double mutant caused wilting symptoms in the *Roq1*‐silenced plants, but not in the control plants (Fig. 8b,c). These findings indicate that Roq1 is involved in the recognition of RipB in *N. benthamiana*.

## Discussion

In this study, we showed that the type III effector RipB from the *R. solanacearum* strain RS1000 acts as an avirulence determinant in *N. benthamiana*. We provided the following lines of evidence. (i) The transient expression of RipB induced defence responses such as ROS bursts and defence gene expression in *N. benthamiana* leaves (Fig. 1). (ii) Deletion of *ripB* abolished the induction of defence responses against *R. solanacearum* RS1002, a Nal^r^ derivative of RS1000, and the Δ*ripB* mutant could grow and cause wilting symptoms in *N. benthamiana* (Fig. 3). (iii) The introduction of *ripB* ^+^ into the Δ*ripB* mutant completely recovered the avirulence phenotype in *N. benthamiana.* On the basis of these findings, we conclude that RipB is a novel Avr effector that triggers ETI responses and restricts the host range of RS1000 to *N. benthamiana.* The RipB effector homologues are widely distributed among several plant pathogenic bacteria, such as *Pseudomonas* and *Xanthomonas* strains (Fig. S4). Among them, HopQ1 from *P. syringae* pv. *tomato* and XopQ from *X. euvesicatoria* have been shown to act as an avirulence determinant in *N. benthamiana* (Schwartz *et al.*, 2015; Wei *et al.*, 2007). Therefore, it is reasonable that RipB from *R. solanacearum* acts as an Avr effector in *N. benthamiana.*

In contrast to RipP1, the *Agrobacterium*‐mediated transient expression of RipB induced chlorosis, but not a typical HR cell death, in *N. benthamiana* leaves, although RipB induced similar ETI responses to RipP1 (Figs 1 and S1). It has been shown that ETI responses induced by Avr proteins are not always accompanied by HR cell death (Bendahmane *et al.*, 1999; Eitas *et al.*, 2008; Yu *et al.*, 1998). The conflict between the HR phenotypes from transient expression (Fig. S1) and *R. solanacearum* inoculation (Fig. 3a,b) might be explained by differences in signalling pathways between HR cell death and other ETI responses. From another viewpoint, it has recently been suggested that the ETI response induced by the RipB homologues XopQ and HopQ1 has severe negative effects on the multiplication of *A. tumefaciens* as well as on *Agrobacterium*‐mediated transient protein expression in *N. benthamiana* (Adlung and Bonas, 2017). Therefore, the chlorosis induced by the transient expression of RipB might be caused by the attenuated transient expression of RipB.

We speculate that RipB is the major avirulence determinant of many Japanese *R. solanacearum* strains in tobacco because the *ripB* single mutant of RS1002 could cause wilting symptoms in *N. benthamiana*, *N. tabacum* and *N. otophora* (Figs 3e,f and 7b–e). On the basis of DNA‐based phylogenetic analyses, *R. solanacearum* strains are divided into phylotypes I, II, III and IV, which consist of a number of sequevars (Fegan and Prior, 2005). RipB is one of the core effectors that are highly conserved among all phylotypes of *R. solanacearum* (Peeters *et al.*, 2013). It would be interesting to examine whether RipB determines avirulence in tobacco among the *R. solanacearum* species complex.

We confirmed that RipP1 and RipAA contribute in part to the avirulence of RS1000 in *Nicotiana* plants (Fig. 4). This finding is in agreement with previous studies revealing the avirulence function of RipP1 and RipAA (Carney and Denny, 1990; Poueymiro *et al.*, 2009). In contrast to RS1000, however, RipP1 and RipAA mainly contribute to the avirulence of GMI1000 in *Nicotiana* plants (Poueymiro *et al.*, 2009). The discrepancy in the contributions of RipP1 and RipAA to avirulence in tobacco between the two strains might be explained by the difference in effector repertoires. GMI1000 was isolated from tomato in French Guyana (South America) and belongs to sequevar 18, which is not distributed in Japan (Boucher *et al.*, 1987; Horita *et al.*, 2014). On the other hand, RS1000 belongs to sequevar 15, to which many Japanese tobacco‐avirulent strains belong (Horita and Tsuchiya, 2012). The *ripP1 ripAA* mutants of several Japanese strains belonging to sequevar 15 or 16 showed no virulence in *N. tabacum* (Chen *et al.*, 2018). Sequevars 15 and 16 are phylogenetically closely related, but are distant from sequevar 18 (Horita *et al.*, 2014). Therefore, the effector repertoire of Japanese tobacco‐avirulent strains might be different from that of GMI1000. It has been reported that *P. syringae* pv. *tomato* DC3000 possesses several type III effectors that can suppress HopQ1‐triggered HR cell death (Wei *et al.*, 2018; Zembek *et al.*, 2018). GMI1000 might possess effectors that suppress RipB‐triggered ETI responses. It is also possible that RipB from GMI1000 induces weaker ETI responses than that from the characterized Japanese strains. Nevertheless, our findings clearly show that the bacterial wilt resistance of *N. benthamiana* is based on the recognition of at least three effector proteins, RipB, RipP1 and RipAA.

It is noteworthy that the tobacco‐virulent *R. solanacearum* strain BK1002 contains mutations in *ripB* and expresses the C‐terminal‐truncated mutant protein of RipB that does not induce ETI responses in *N. benthamiana* (Fig. 5a–d). This finding strongly supports the avirulence activity of RipB and suggests that the naturally occurring *ripB* mutation that disrupts the avirulence activity expanded the host range of *R. solanacearum* to *Nicotiana* plants in the field. It has been shown that *hopQ1* is missing in the genome of the tobacco pathogen *P. syringae* pv. *tabaci* (Ferrante *et al.*, 2009). Our work provides new evidence of fine‐tuning of a pathogen effector repertoire during pathoadaptation in *Nicotiana* plants. The microscopy analysis using GFP fusion proteins revealed that the C‐terminal deletion alters the subcellular localization of RipB in plant cells (Fig. 5e). The deletion of the C‐terminal domain itself or the altered protein localization might abolish the recognition of RipB in *N. benthamiana*.

Recent research revealed that the TIR‐NLR protein Roq1 recognizes XopQ and HopQ1 in *N. benthamiana* (Schultink *et al.*, 2017). In this study, we observed that *R. solanacearum* RS1002 is avirulent in *Nicotiana* plants that contain an intact *Roq1*, but not in *N. sylvestris* that contains an aberrant copy of *Roq1* (Fig. 7a). The *ripP1 ripAA* mutant of RS1002 that expresses RipB, but not RipP1 or RipAA, became virulent in the *Roq1*‐silenced *N. benthamiana* plants (Fig. 8). These findings indicate that Roq1 is involved in the recognition of RipB in *Nicotiana* plants. To date, the RPS4 and RRS1 NLR protein pair that recognize RipP2 have been reported in *A. thaliana* (Deslandes *et al.*, 2003; Narusaka *et al.*, 2009). As far as we know, Roq1 is the second R protein found to be involved in the recognition of a *R. solanacearum* effector protein. Although the distribution of RipP2 is limited, RipB is widely distributed among the *R. solanacearum* species complex (Peeters *et al.*, 2013). Our work suggests that *Roq1* is a useful genetic resource for breeding durable resistance in crops against bacterial wilt.

## Experimental Procedures

### Plant and bacterial growth conditions

*Nicotiana* plants were grown in a controlled environment room as described previously (Nakano *et al.*, 2017). The bacterial strains used in this study are listed in Table S1. The growth conditions, media and antibiotics used for the *Escherichia coli*, *R. solanacearum* and *A. tumefaciens* strains were described previously (Mukaihara *et al.*, 2004; Nakano *et al.*, 2017).

### Generation of *R. solanacearum* mutants

For the generation of Δ*ripB* mutants, each fragment (0.6 kb) upstream and downstream of the *ripB* coding region was inserted into the plasmid pK18*mobsacB* (Schäfer *et al.*, 1994) using an In‐Fusion HD cloning kit (Takara, Shiga, Japan) to yield pK18*mobsacB*‐Δ*ripB*. For the generation of Δ*ripP1* mutants, each fragment (0.7 kb) upstream containing a part of the *ripP1*‐coding region and downstream of *ripP1* was amplified and joined by PCR. The resultant fragment carrying a Δ*ripP1* fragment was inserted into the *Sma*I‐*Hin*dIII sites of pK18*mobsacB* to yield pK18*mobsacB*‐Δ*ripP1*. For the generation of *ripAA* mutants, a 2.8‐kb fragment containing the entire *ripAA* coding region was inserted into the *Sma*I‐*Xba*I sites of pK18*mobsacB*. The resultant plasmid was digested with *Bam*HI, and the gentamicin resistance (Gm^r^) cassette derived from pMS252 (Becker *et al.*, 1995) was inserted into the *Bam*HI site in the *ripAA* coding region to yield pK18*mobsacB*‐*ripAA*::Gm^r^. These pK18*mobsacB*‐derived plasmids were used to generate *R. solanacearum* mutants by the marker‐exchange method using *E. coli* S17‐1 (Schäfer *et al.*, 1994). The primer sets used in this study are listed in Table S2.

For complementation experiments of the Δ*ripB* mutant, each gene fragment of the entire *ripB* coding region containing its promoter region and its derivatives was inserted into the plasmid pARO191 (Parke, 1990). The resultant plasmids were integrated into the genome of the Δ*ripB* mutant by a homologous recombination at the native locus through conjugation with S17‐1.

### *Agrobacterium*‐mediated transient expression (agroinfiltration)

The entire coding region of RS1000 effector genes was inserted into the *Spe*I site of the pEL2Ω‐MCS vector under the control of the constitutive promoter (Nahar *et al.*, 2014). *Agrobacterium tumefaciens* cells harbouring the resultant plasmids were suspended in infiltration buffer [10 mM MgCl_2_, 10 mM MES (pH 5.6)] supplemented with 150 μM acetosyringone. The inoculum preparations were spectrometrically adjusted to OD_600_ = 0.5 and incubated at 30 °C for 3 h with shaking before infiltration.

For the observation of subcellular localization, *ripB* and its derivative genes were inserted into the *Bam*HI site of the pEL2Ω‐GFP vector (Mukaihara *et al.*, 2016). The resultant plasmids were used for *Agrobacterium*‐mediated transient expression in *N. benthamiana* leaves. Leaves were infiltrated with a solution of 25 μg/mL DAPI (Sigma, St Louis, MO, USA) 1 day after the agroinfiltration. The fluorescence of GFP and DAPI was observed 2 h after the infiltration of DAPI under a laser scanning microscope (FV1200, Olympus, Tokyo, Japan).

### Measurement of ROS and ion leakage

ROS levels were measured using a chemiluminescence probe (L‐012, Wako, Osaka, Japan) as described previously (Nakano *et al.*, 2015). Leaves were infiltrated with 0.5 mM L‐012 solution (10 mM MOPS‐KOH pH 7.4), and chemiluminescence was detected using a luminescence imaging system (ChemiDoc MP, Bio‐Rad, Hercules, CA, USA).

The severity of cell death was quantified by the degree of electrolyte leakage from leaves. A suspension of *R. solanacearum* was infiltrated into the leaves of *N. benthamiana* at 1 × 10^8^ CFU/mL using a needleless syringe. Leaf disks (8 mm in diameter) were immersed in 1 mL of water for 2 h with gentle shaking. The ion conductivity of water was measured using a conductivity meter (LAQUAtwin, Horiba, Kyoto, Japan).

### Real‐time PCR

Real‐time PCR was performed as described previously (Nakano *et al.*, 2013). Briefly, a suspension of *R. solanacearum* was infiltrated into the leaves of *N. benthamiana* at 5 × 10^7^ CFU/mL using a needleless syringe. Total RNA was extracted from leaves using an RNeasy Plant Mini Kit (Qiagen, Hilden, Germany), and cDNA was synthesized using a High Capacity cDNA reverse transcription kit (Applied Biosystems, Foster, CA, USA). Quantitative PCR was performed using a Power SYBR Green PCR master mix (Applied Biosystems). Expression levels of target genes were normalized to those of multiple endogenous control genes such as *NbEF1α* and *NbF‐box*. Gene‐specific primers are listed in Table S2.

### VIGS

Gene silencing in *N. benthamiana* was performed using the TRV‐based vectors pTRV1 and pTRV2 (Liu *et al.*, 2002). The fragments of target genes were inserted into the *Bam*HI site of pTRV2 using an In‐Fusion HD cloning kit. *Agrobacterium tumefaciens* cells harbouring the resultant plasmids were suspended in infiltration buffer supplemented with 150 μM acetosyringone. The inoculum preparations were spectrometrically adjusted to OD_600_ = 1.0 and incubated at 30 °C for 3 h with shaking. A mixture of *A. tumefaciens* cells harbouring pTRV1 and pTRV2 derivatives (1:1 ratio) was infiltrated into the leaves. Three‐ to 4‐week‐old plants after VIGS were used for plant assays.

### Bacterial virulence assay

The virulence of* R. solanacearum* in *Nicotiana* plants was assayed as described previously (Nakano and Mukaihara, 2018; Nakano *et al.*, 2017). For measuring bacterial growth, a suspension of *R. solanacearum* was infiltrated into the leaves of *N. benthamiana* at 1 × 10^4^ CFU/mL. Leaf disks were taken from the inoculated leaves and homogenized in water. Serial dilutions of the homogenate were spread on BG plates containing nalixidic acid. For pathogenicity tests, a suspension of *R. solanacearum* was infiltrated into the leaves of *N. benthamiana* at 1 × 10^8^ CFU/mL. The severity of wilting symptoms was evaluated daily using the following disease index: 0, no wilting; 1, 1–33% wilted leaves; 2, 34–66% wilted leaves; 3, 67–99% wilted leaves; 4, completely wilted.

### Protein extraction and immunoblotting

Leaf disks (60 mg) were collected, frozen in liquid nitrogen and ground to a fine powder. Proteins were extracted in 60 μL of extraction buffer [0.35 M Tris‐HCl (pH 6.8), 30% glycerol, 10% SDS, 0.6 M DTT, 0.012% bromophenol blue]. Total protein (10 μL) was separated by 10% SDS‐PAGE. Separated proteins were transferred onto a membrane and incubated with an HRP‐conjugated anti‐GFP antibody (1:5000; Miltenyi Biotec, Bergisch Gladbach, Germany). Immunodetection was performed using an ECL Prime western blotting detection reagent (GE Healthcare, Marlborough, MA, USA).

## Supporting information


**Fig. S1** Effect of prolonged expression of RipB in *Nicotiana benthamiana*. Leaves were infiltrated with *Agrobacterium tumefaciens* harbouring the binary vector expressing RipB, RipP1 or green fluorescent protein (GFP). Photographs were taken 4 and 7 days after agroinfiltration. The experiment was repeated three times with similar results and representative results are shown.

**Fig. S2** Expressions of target genes in the virus‐induced gene silencing plants. Seedlings of *Nicotiana benthamiana *were coinfiltrated with *Agrobacterium tumefaciens* harbouring pTRV1 and that harbouring pTRV2 (TRV:EV), pTRV2:NbCOI1 (TRV:NbCOI1), pTRV2:NbEIN2 (TRV:NbEIN2), pTRV2:NbICS1 (TRV:NbICS1), pTRV2:NbSGT1 (TRV:NbSGT1) or pTRV2:NbRbohB (TRV:NbRbohB). Total RNA was isolated from the leaves of the control and the *NbCOI1*‐, *NbEIN2*‐, *NbICS1*‐,* NbSGT1*‐ and *NbRbohB*‐silenced plants, and gene expression was analysed by semiquantitative RT‐PCR. The housekeeping gene *NbACT* was used as the endogenous control.

**Fig. S3** Virulence test using the stem inoculation method. Stems of *Nicotiana benthamiana* were inoculated with the suspensions of *R. solanacearum* RS1002 (WT) and the Δ*ripP1* *ripAA*::Gm^r^, Δ*ripB* and Δ*ripB* (*ripB* ^+^) mutants. (a) Disease development in plants inoculated with *R. solanacearum* mutants. The severity of wilting symptoms was scored daily using the disease index. (b) Disease symptoms in plants in (a). Photographs were taken 7 days after inoculation. All experiments were repeated three times with similar results and representative results are shown.

**Fig. S4** Amino acid sequence comparison among RipB homologues. Protein sequences were aligned using the Clustal Omega program. Identical amino acid residues are shown in black. Black arrowheads denote the different residues between RipB and RipB_BK_. Accession numbers: RipB (LC459954) and RipB_BK_ (LC459955) from *Ralstonia solanacearum* strains RS1002 and BK1002, respectively; HopQ1_Pst_ (AAO54411) from *Pseudomonas syringae* pv. *tomato*; HopQ1_Psp_ (AAZ37975) from *P. savastanoi* pv. *phaseolicola*; XopQ_Xe_ (CAJ26169) from *Xanthomonas euvesicatoria*; XopQ_Xoo_ (AOS04290) from *X*.* oryzae* pv. *oryzae*; XopQ_Xaj_ (OAH88625) from *X*.* arboricola* pv. *juglandis*; XopQ_Xg_ (EGD20378) from *X*.* gardneri.*

**Fig. S5** Transient expression of green fluorescent protein (GFP)‐tagged RipB in *Nicotiana benthamiana*. Leaves were infiltrated with *Agrobacterium tumefaciens* harbouring the binary vector expressing the C‐terminal GFP‐tagged RipB, RipB^ΔCter^ or RipB_BK_, or GFP alone. (a) Immunoblot analysis of RipB‐GFP fusions. Total protein was extracted from the leaves 1 day after agroinfiltration and subjected to immunoblot analysis using an anti‐GFP antibody. The membrane was stained with Ponceau S as the loading control. (b) Morphological features of leaves expressing RipB‐GFP fusions. Photograph was taken 1 week after agroinfiltration. All experiments were repeated three times with similar results and representative results are shown.

**Fig. S6** Effect of prolonged expression of RipB in *Nicotiana* plants. Leaves of *N. tabacum *(a), *N. otophora *(b) and *N. sylvestris *(c) were infiltrated with *Agrobacterium tumefaciens* harbouring the binary vector expressing RipB or the green fluorescent protein (GFP). Photographs were taken 7 days after agroinfiltration. All experiments were repeated three times with similar results and representative results are shown.

**Fig. S7** Expression of *Roq1* in the virus‐induced gene silencing plants. Seedlings of *Nicotiana benthamiana *were coinfiltrated with *Agrobacterium tumefaciens* harbouring pTRV1 and that harbouring pTRV2 (TRV:EV) or pTRV2:Roq1 (TRV:Roq1). Total RNA was isolated from leaves of the control and *Roq1*‐silenced plants, and gene expression was analysed by semiquantitative RT‐PCR. The housekeeping gene *NbACT* was used as the endogenous control.

**Table S1** Bacterial strains used in this study.

**Table S2** Primer sets used in this study.
