# Supplementary figures and images for: The type III effector RipB from Ralstonia solanacearum RS1000 acts as a major avirulence factor in Nicotiana benthamiana and other Nicotiana species
Source: Mol Plant Pathol. 2019 Jun 20;20(9):1237–51. doi: 10.1111/mpp.12824 (PMC6715614; doi:10.1111/mpp.12824)

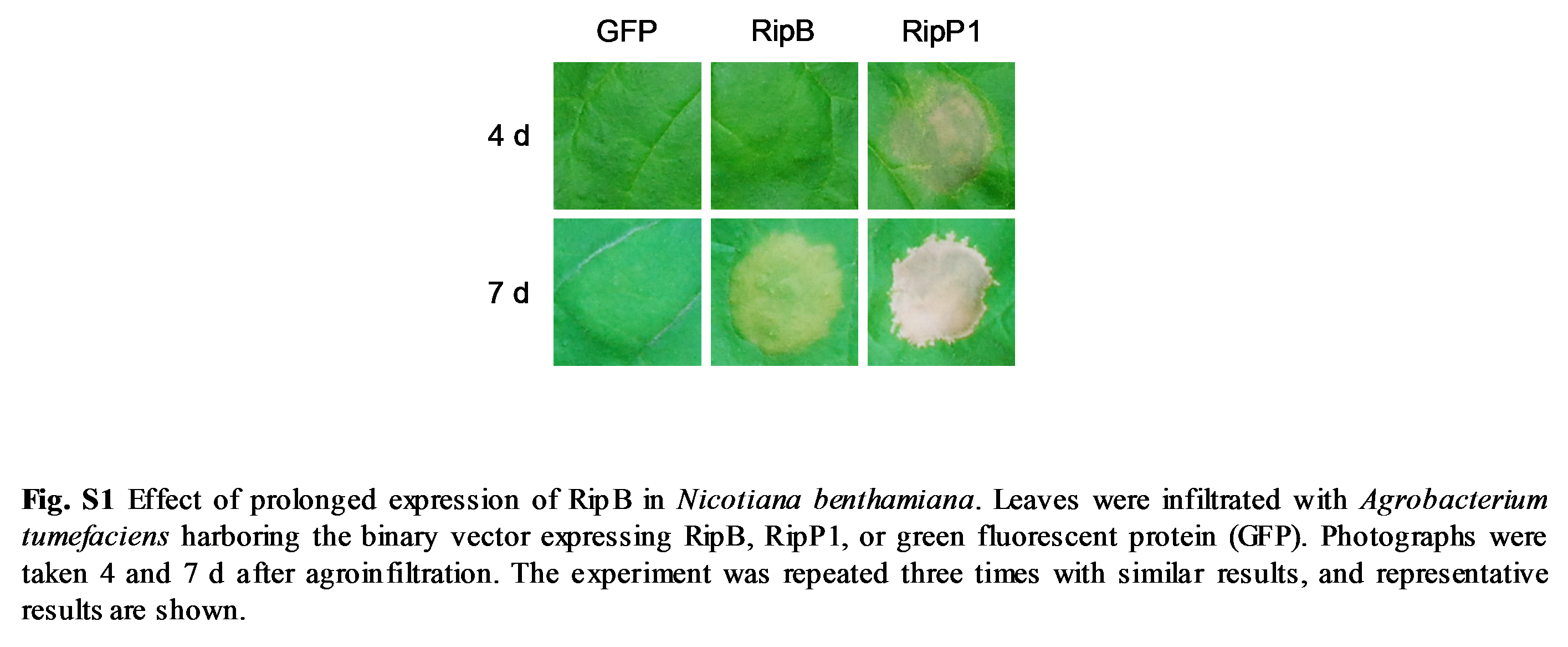

Supplement: Supplementary file 1 — Fig. S1 Effect of prolonged expression of RipB in Nicotiana benthamiana. Leaves were infiltrated with Agrobacterium tumefaciens harbouring the binary vector expressing RipB, RipP1 or green fluorescent protein (GFP). Photographs were taken 4 and 7 days after agroinfiltration. The experiment was repeated three times with similar results and representative results are shown. [file MPP-20-1237-s001.tif]

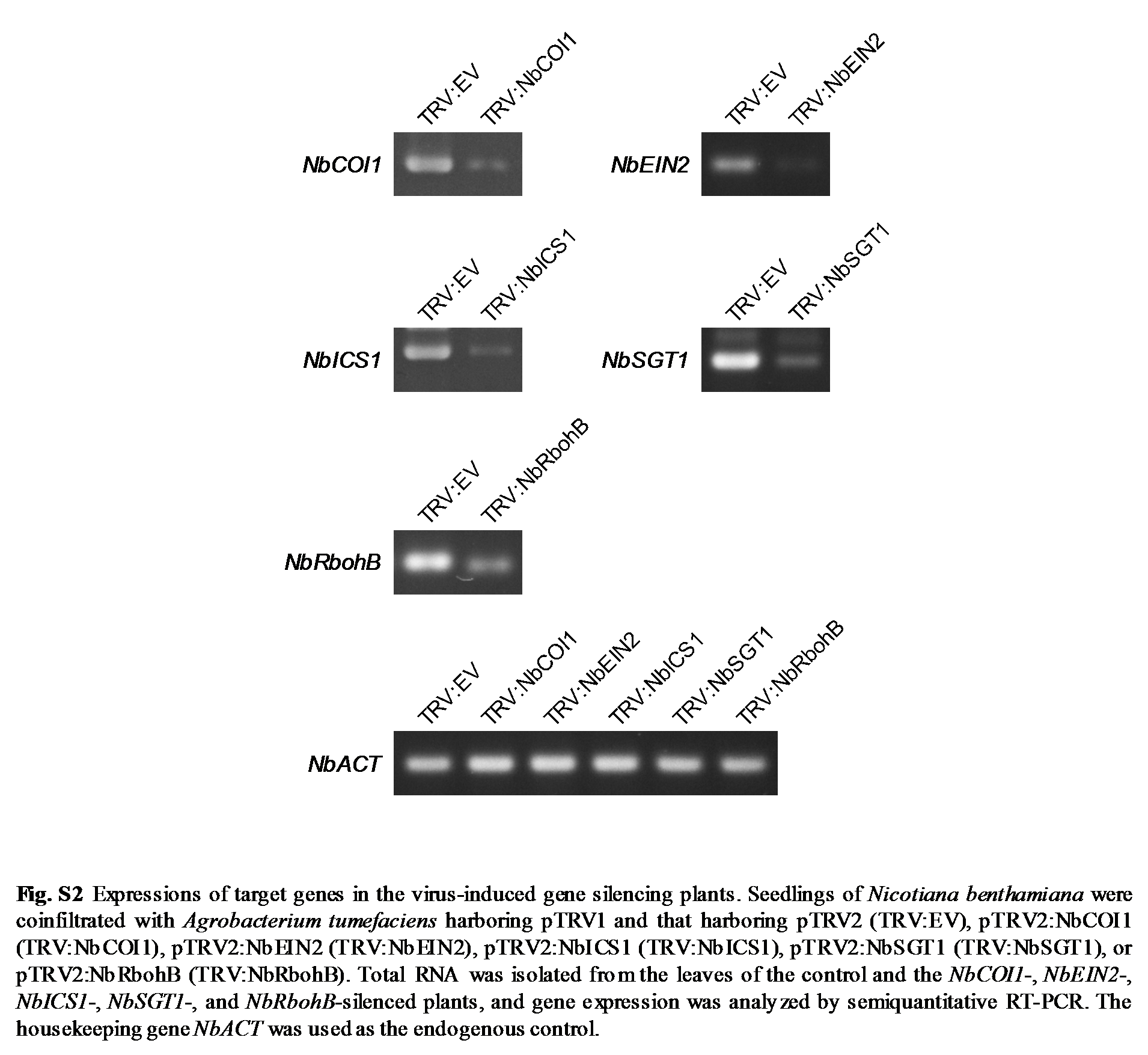

Supplement: Supplementary file 2 — Fig. S2 Expressions of target genes in the virus‐induced gene silencing plants. Seedlings of Nicotiana benthamiana were coinfiltrated with Agrobacterium tumefaciens harbouring pTRV1 and that harbouring pTRV2 (TRV:EV), pTRV2:NbCOI1 (TRV:NbCOI1), pTRV2:NbEIN2 (TRV:NbEIN2), pTRV2:NbICS1 (TRV:NbICS1), pTRV2:NbSGT1 (TRV:NbSGT1) or pTRV2:NbRbohB (TRV:NbRbohB). Total RNA was isolated from the leaves of the control and the NbCOI1‐, NbEIN2‐, NbICS1‐, NbSGT1‐ and NbRbohB‐silenced plants, and gene expression was analysed by semiquantitative RT‐PCR. The housekeeping gene NbACT was used as the endogenous control. [file MPP-20-1237-s002.tif]

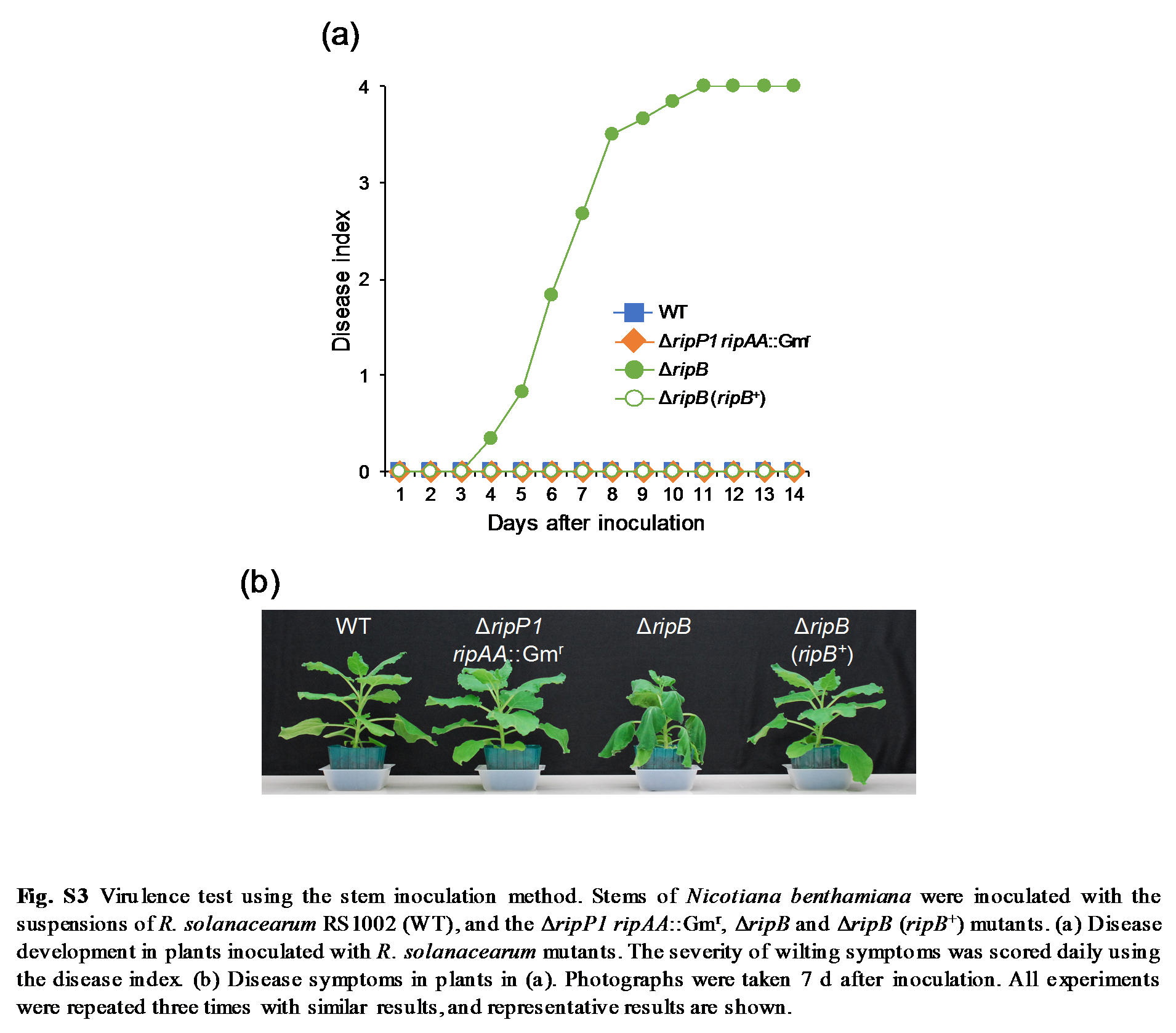

Supplement: Supplementary file 3 — Fig. S3 Virulence test using the stem inoculation method. Stems of Nicotiana benthamiana were inoculated with the suspensions of R. solanacearum RS1002 (WT) and the ΔripP1 ripAA::Gmr, ΔripB and ΔripB (ripB +) mutants. (a) Disease development in plants inoculated with R. solanacearum mutants. The severity of wilting symptoms was scored daily using the disease index. (b) Disease symptoms in plants in (a). Photographs were taken 7 days after inoculation. All experiments were repeated three times with similar results and representative results are shown. [file MPP-20-1237-s003.tif]

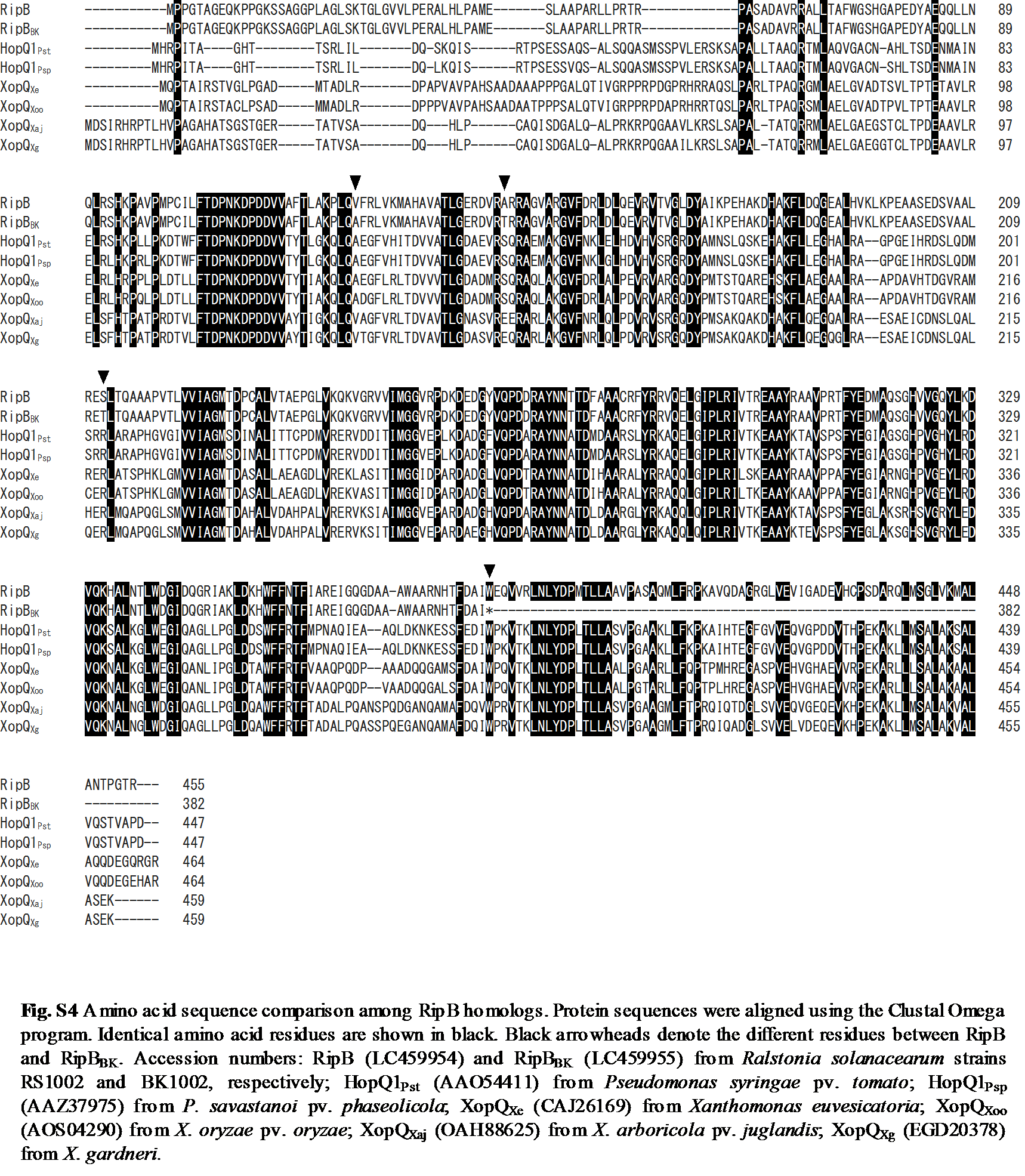

Supplement: Supplementary file 4 — Fig. S4 Amino acid sequence comparison among RipB homologues. Protein sequences were aligned using the Clustal Omega program. Identical amino acid residues are shown in black. Black arrowheads denote the different residues between RipB and RipBBK. Accession numbers: RipB (LC459954) and RipBBK (LC459955) from Ralstonia solanacearum strains RS1002 and BK1002, respectively; HopQ1Pst (AAO54411) from Pseudomonas syringae pv. tomato; HopQ1Psp (AAZ37975) from P. savastanoi pv. phaseolicola; XopQXe (CAJ26169) from Xanthomonas euvesicatoria; XopQXoo (AOS04290) from X. oryzae pv. oryzae; XopQXaj (OAH88625) from X. arboricola pv. juglandis; XopQXg (EGD20378) from X. gardneri. [file MPP-20-1237-s004.tif]

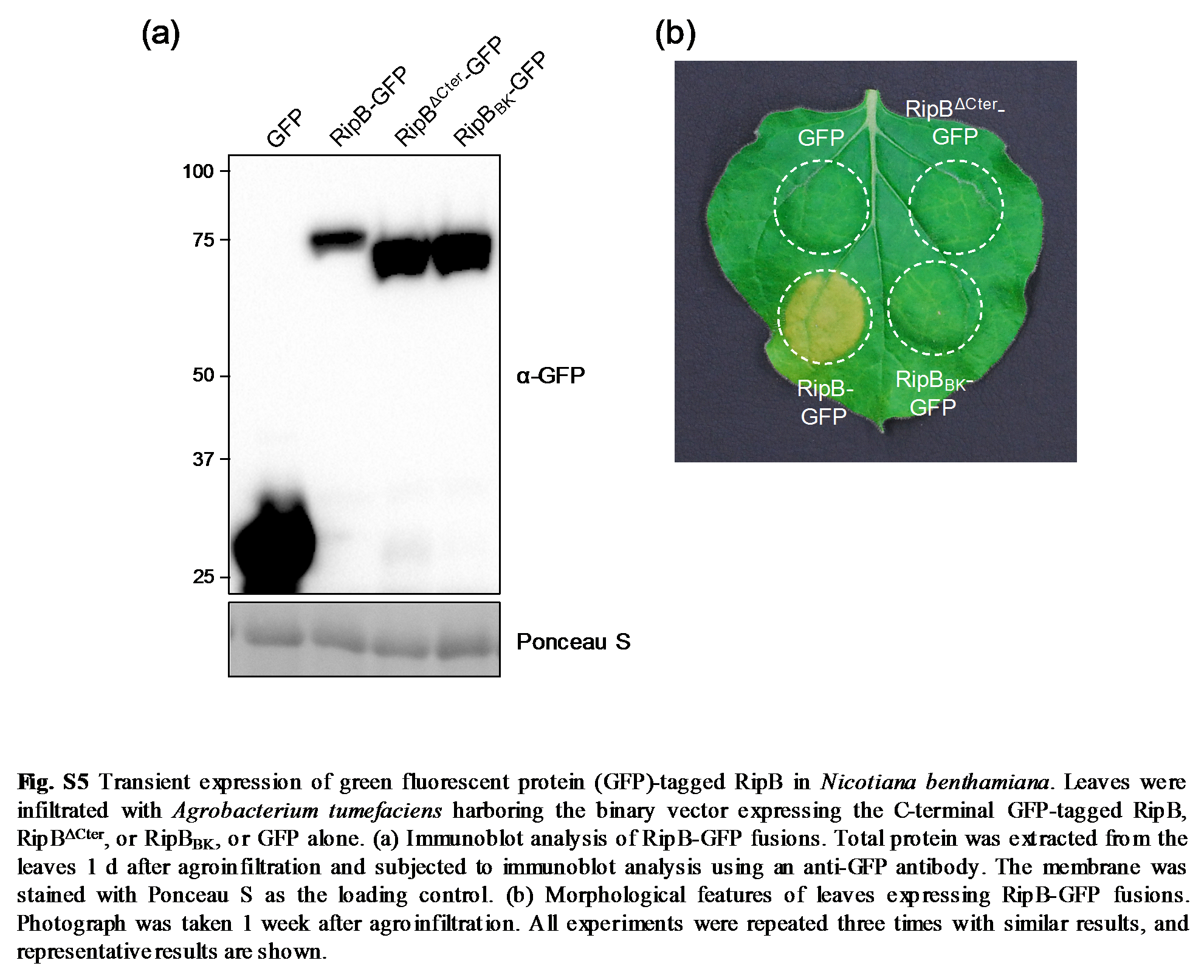

Supplement: Supplementary file 5 — Fig. S5 Transient expression of green fluorescent protein (GFP)‐tagged RipB in Nicotiana benthamiana. Leaves were infiltrated with Agrobacterium tumefaciens harbouring the binary vector expressing the C‐terminal GFP‐tagged RipB, RipBΔCter or RipBBK, or GFP alone. (a) Immunoblot analysis of RipB‐GFP fusions. Total protein was extracted from the leaves 1 day after agroinfiltration and subjected to immunoblot analysis using an anti‐GFP antibody. The membrane was stained with Ponceau S as the loading control. (b) Morphological features of leaves expressing RipB‐GFP fusions. Photograph was taken 1 week after agroinfiltration. All experiments were repeated three times with similar results and representative results are shown. [file MPP-20-1237-s005.tif]

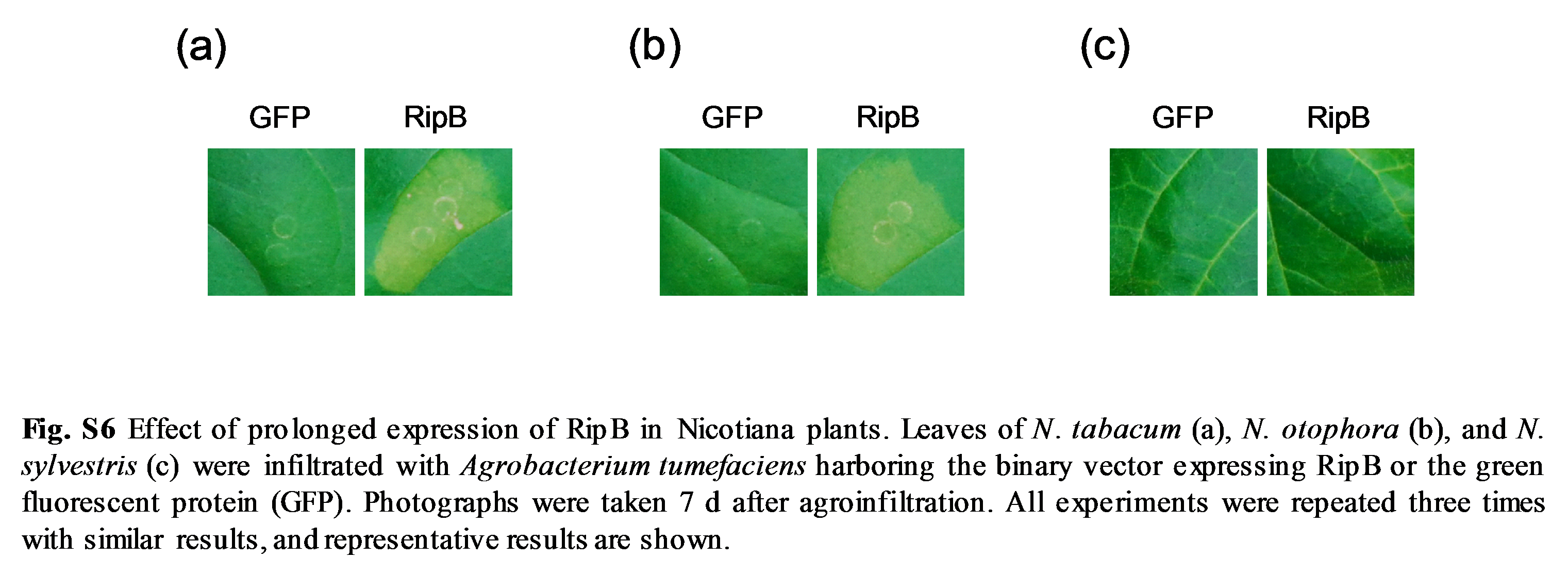

Supplement: Supplementary file 6 — Fig. S6 Effect of prolonged expression of RipB in Nicotiana plants. Leaves of N. tabacum (a), N. otophora (b) and N. sylvestris (c) were infiltrated with Agrobacterium tumefaciens harbouring the binary vector expressing RipB or the green fluorescent protein (GFP). Photographs were taken 7 days after agroinfiltration. All experiments were repeated three times with similar results and representative results are shown. [file MPP-20-1237-s006.tif]

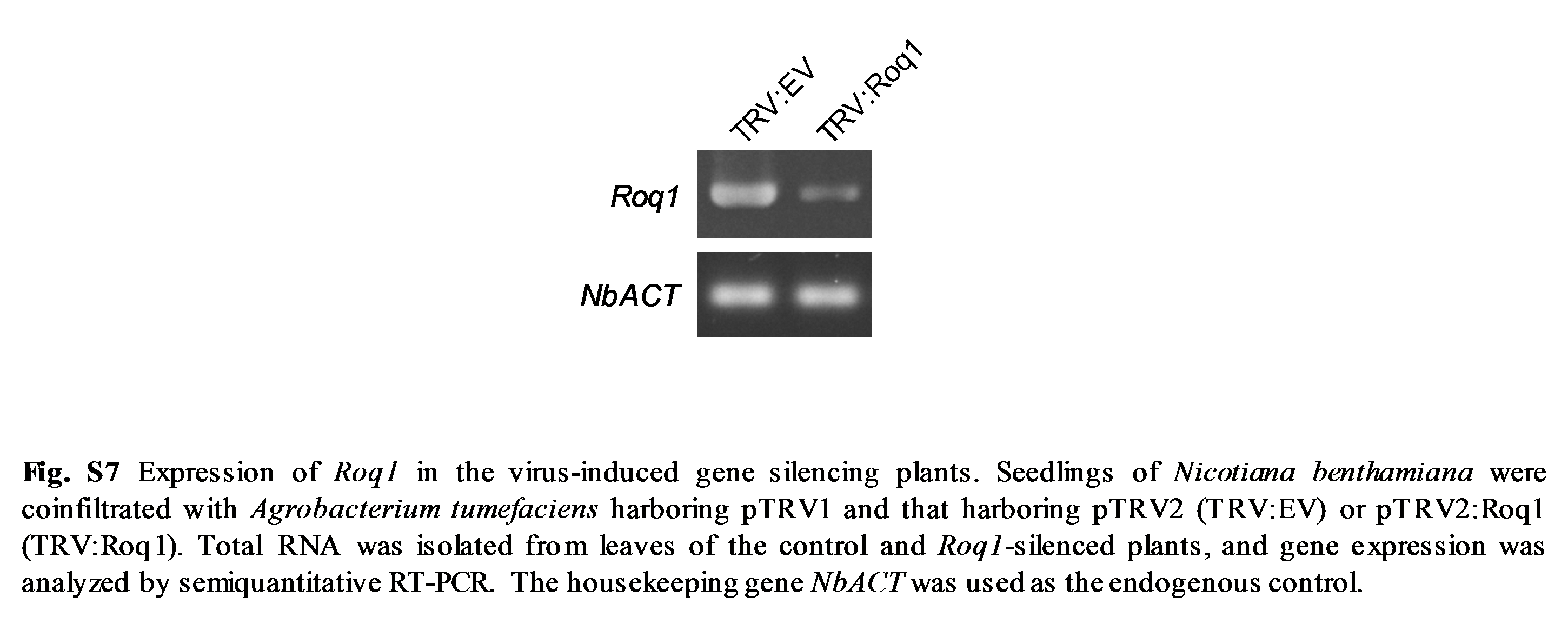

Supplement: Supplementary file 7 — Fig. S7 Expression of Roq1 in the virus‐induced gene silencing plants. Seedlings of Nicotiana benthamiana were coinfiltrated with Agrobacterium tumefaciens harbouring pTRV1 and that harbouring pTRV2 (TRV:EV) or pTRV2:Roq1 (TRV:Roq1). Total RNA was isolated from leaves of the control and Roq1‐silenced plants, and gene expression was analysed by semiquantitative RT‐PCR. The housekeeping gene NbACT was used as the endogenous control. [file MPP-20-1237-s007.tif]
