# Supplementary material for: The type III effector RipB from Ralstonia solanacearum RS1000 acts as a major avirulence factor in Nicotiana benthamiana and other Nicotiana species
Source: Mol Plant Pathol. 2019 Jun 20;20(9):1237–51. doi: 10.1111/mpp.12824 (PMC6715614; doi:10.1111/mpp.12824)
Supplement: Supplementary file 8 — Table S1 Bacterial strains used in this study. [file MPP-20-1237-s008.docx]

**TABLE S1** Bacterial strains used in this study.

| Strain | Relevant genotype | Reference |
| --- | --- | --- |
| *Escherichia coli* |  |  |
| S17-1 | *E. coli* K12 *thi* *pro* *hsdR*^−^ *hsdM*^+^ *recA* [chr::RP4-2-Tc::Mu-Km::Tn*7*] | Simon *et al*., 1983 |
|  |  |  |
| *Ralstonia solanacearum* |  |  |
| RS1002 | RS1000 Nal^r^ (phylotype I, biovar 4, sequevar 15) | Mukaihara *et al*., 2004 |
| RS1650 | RS1002 *ripAA*::Gm^r^ | This study |
| RS1660 | RS1002 Δ*ripP1* | This study |
| RS1661 | RS1002 Δ*ripP1* *ripAA*::Gm^r^ | This study |
| RS1712 | RS1002 Δ*ripB* | This study |
| RS1713 | RS1712 Δ*ripB* chr::pARO-ripB^+^ | This study |
| RS1714 | RS1712 Δ*ripB* chr::pARO-ripB^ΔCter^ | This study |
| RS1715 | RS1712 Δ*ripB* chr::pARO-ripB_BK_ | This study |
| RS1716 | RS1002 Δ*ripB* *ripAA*::Gm^r^ | This study |
| RS1717 | RS1002 Δ*ripB* Δ*ripP1* | This study |
| RS1718 | RS1002 Δ*ripB* Δ*ripP1* *ripAA*::Gm^r^ | This study |
| BK1002 | BK1000 Nal^r^ (phylotype I, biovar 3, sequevar 34) | Tamura *et al*., 2002 |
|  |  |  |
| *Agrobacterium tumefaciens* |  |  |
| GV3101 | Gm^r^ Rif^r^ | Lamblin *et al*., 2001 |

**Lamblin F, Saladin G, Dehorter B, Cronier D, Grenier E *et al*.** (2001) Overexpression of a heterologous *sam* gene encoding S-adenosylmethionine synthetase in flax (*Linum usitatissimum*) cells: consequences on methylation of lignin precursors and pectins. *Physiol Plant*. 112: 223–232.

**Mukaihara T, Tamura N, Murata Y, Iwabuchi M.** (2004) Genetic screening of Hrp type III-related pathogenicity genes controlled by the HrpB transcriptional activator in *Ralstonia solanacearum*. *Mol Microbiol*. 54(4): 863–875.

**Simon R, Priefer U, Pühler A.** (1983) A broad host range mobilization system for *in vivo* genetic engineering: Transposon mutagenesis in Gram-negative bacteria. *Nature Biotechnology*. 1: 784–791.

**Tamura N, Murata Y, Mukaihara T.** (2002) A somatic hybrid between *Solanum integrifolium* and *Solanum violaceum* that is resistant to bacterial wilt caused by *Ralstonia solanacearum.* *Plant Cell Rep*. 21(4): 353–358.
