# Supplementary material for: The type III effector RipB from Ralstonia solanacearum RS1000 acts as a major avirulence factor in Nicotiana benthamiana and other Nicotiana species
Source: Mol Plant Pathol. 2019 Jun 20;20(9):1237–51. doi: 10.1111/mpp.12824 (PMC6715614; doi:10.1111/mpp.12824)
Supplement: Supplementary file 9 — Table S2 Primer sets used in this study. [file MPP-20-1237-s009.docx]

**TABLE S2** Primer sets used in this study.

| Target gene | Purpose | Sequence (5’-3’) |
| --- | --- | --- |
| *ripB* | Cloning / Transient expression | GCTCTAGAATGCCGCCAGGCACAGCCGGCG |
|  |  | GGACTAGTTCAGCGCGTACCCGGCGTGTTC |
| *ripP1* | Cloning / Transient expression | GGTCTAGAATGAGAAGACTATTTAGAGCAT |
|  |  | GGACTAGTTCACGACTCCAGGGCATGTCGA |
| *ripB* | Transient expression / Localization | TACATCTAGAGGATCATGCCGCCAGGCACAGC |
|  |  | TGCTCACCATGGATCCGCGCGTACCCGGCGTG |
| *ripB^ΔCter^* / *ripB_BK_* | Transient expression / Localization | TACATCTAGAGGATCATGCCGCCAGGCACAGC |
|  |  | TGCTCACCATGGATCCGATCGCGTCGAACGTGTG |
| *NbCOI1* | VIGS | GCCTCCATGGGGATCGGACATCCTTTAGAGAACTC |
|  |  | GCTCGGTACCGGATCGGCAAAGGGATGCCTTGA |
| *NbEIN2* | VIGS | GCCTCCATGGGGATCCCATATCCCATGGAATTGTA |
|  |  | GCTCGGTACCGGATCCCAGTATGCGGATTCCAG |
| *NbICS1* | VIGS | GCCTCCATGGGGATCGCCTCACTACAAATGCTG |
|  |  | GCTCGGTACCGGATCGCTGCAACAATTGCATGGG |
| *NbRbohB* | VIGS | GCCTCCATGGGGATCCCTTAGACTCCTTATTCATAC |
|  |  | GCTCGGTACCGGATCGCGAGATTTAACATTCCTGA |
| *Roq1* | VIGS | GCCTCCATGGGGATCCTAGGGAGACTTACAAGTTGG |
|  |  | GCTCGGTACCGGATCCATCCATCTGTTGGGTTTCTC |
| *NbCOI1* | RT-PCR | GGATAGAGGATTGGAAGTGCTTGG |
|  |  | GCCAAAGCTAATGCACGTTCAC |
| *NbEIN2* | RT-PCR | CAGAAGCGAAGTCCAAGAAACTGG |
|  |  | GGCTTGTTCGTACGTAGATCGAC |
| *NbICS1* | RT-PCR | GCAATCCGATTTGATGCAACAGC |
|  |  | CTGTAGGATACCCACAAACTGCTGG |
| *NbSGT1* | RT-PCR | GCTGCCAGACCAAAATACAGGCATG |
|  |  | CTCTTCTGGTGTCCTCATCGGCATC |
| *NbRbohB* | RT-PCR | GAGATAATAGGAAATGACAGAGCGTCG |
|  |  | CTCCGAGCAAGCGCATCATATAGC |
| *Roq1* | RT-PCR | GCTCTTGTGATGCATTGGTG |
|  |  | CTGGTAAACTCCCCAGTTTCTCAC |
| *NbACT* | RT-PCR | GGGACTGGAATGGTCAAGGC |
|  |  | GCATAAAGGGACAGAACGGC |
| *NbPR1* | qRT-PCR | CTGAGGGAAGTGGCGATTTC |
|  |  | CTCATCGACCCACATCTCAAC |
| *NbHIN1* | qRT-PCR | AGCCTATTATGGCCCTTCCATT |
|  |  | GGCCGTGGCGGTGATA |
| *NbHsr203J* | qRT-PCR | CCACCCCATGACGACTTCA |
|  |  | TTTCGTCGGCAACTATATCTTTGA |
| *NbEF1α* | qRT-PCR | CCCAAGAGGCCCTCAGACA |
|  |  | CACACGACCAACAGGGACAGT |
| *NbF-box* | qRT-PCR | GGCACTCACAAACGTCTATTTC |
|  |  | ACCTGGGAGGCATCCTGCTTAT |
| *ripAA* | Deletion mutant | AGATCAAGGCGCTGATCCAC |
|  |  | CCCACGCCATCGGTATAGGG |
| *ripP1* upstream | Deletion mutant | GGCCCGGGCTAAGCTGGATGTAAGTTCGTG |
|  |  | CAAAACCACCCGCGAGTCAGTGATGCCGGACATCTGCTGC |
| *ripP1* downstream | Deletion mutant | AGCAGATGTCCGGCATCACTGACTCGCGGGTGGTTTTGGT |
|  |  | GGAAGCTTGACACGCCTGAAGCGCATCGCC |
| *ripB* upstream | Deletion mutant | CGACTCTAGAGGATCCAAGCTAACTGCCAGAACGAT |
|  |  | CGGATCGGCGCCGGGGCTGTTGTGTGGTCGCAGCG |
| *ripB* downstream | Deletion mutant | CGACCACACAACAGCCCCGGCGCCGATCCGCCGGC |
|  |  | CGGTACCCGGGGATCCGCCGTGCTGCAGGACGCC |
| *ripB* | Complementation | CGACTCTAGAGGATCCAGGCCGACCGTGAACAGAATC |
|  |  | CGGTACCCGGGGATCGACATCGTGATCGAGGCCGCC |
| *ripB^ΔCter^* / *ripB_BK_* | Complementation | CGACTCTAGAGGATCCAGGCCGACCGTGAACAGAATC |
|  |  | CGGTACCCGGGGATCTCAGATCGCGTCGAACGTGTG |
